# Identification of a Region in the Common Amino-terminal Domain of Hendra Virus P, V, and W Proteins Responsible for Phase Transition and Amyloid Formation

**DOI:** 10.3390/biom11091324

**Published:** 2021-09-07

**Authors:** Edoardo Salladini, Frank Gondelaud, Juliet F. Nilsson, Giulia Pesce, Christophe Bignon, Maria Grazia Murrali, Roxane Fabre, Roberta Pierattelli, Andrey V. Kajava, Branka Horvat, Denis Gerlier, Cyrille Mathieu, Sonia Longhi

**Affiliations:** 1 Laboratory Architecture et Fonction des Macromolécules Biologiques (AFMB), UMR 7257, Centre National de la Recherche Scientifique (CNRS), Aix Marseille University, CEDEX 9, 13288 Marseille, France; edoardo.salladini@gmail.com (E.S.); frank.gondelaud@univ-amu.fr (F.G.); juliet.nilsson@univ-amu.fr (J.F.N.); giulia.pesce@univ-amu.fr (G.P.); christophe.bignon@univ-amu.fr (C.B.); 2 Magnetic Resonance Center (CERM) and Department of Chemistry “Ugo Schiff”, University of Florence, 50019 Sesto Fiorentino, Italy; mgmurrali@gmail.com (M.G.M.); roberta.pierattelli@unifi.it (R.P.); 3 Centre d’Immunologie de Marseille-Luminy (CIML), CNRS, Institut National de la Santé et de la Recherche Médicale (INSERM), Aix Marseille University, CEDEX 9, 13288 Marseille, France; fabre@ciml.univ-mrs.fr; 4 Centre de Recherche en Biologie Cellulaire de Montpellier, UMR 5237, CNRS, Université Montpellier, 34293 Montpellier, France; andrey.kajava@crbm.cnrs.fr; 5 Team Immunobiology of the Viral Infections, Centre International de Recherche en Infectiologie (CIRI), Université de Lyon, INSERM, U1111, CNRS, UMR 5308, Université Claude Bernard Lyon 1, Ecole Normale Supérieure de Lyon, 69007 Lyon, France; branka.horvat@inserm.fr (B.H.); denis.gerlier@inserm.fr (D.G.); cyrille.mathieu@inserm.fr (C.M.)

**Keywords:** Hendra virus, V protein, intrinsically disordered proteins, amyloids, fibrils, phase separation and transitions, TEM, SAXS, CR binding assays, Hsp70

## Abstract

Henipaviruses are BSL-4 zoonotic pathogens responsible in humans for severe encephalitis. Their V protein is a key player in the evasion of the host innate immune response. We previously showed that the *Henipavirus* V proteins consist of a long intrinsically disordered N-terminal domain (NTD) and a β-enriched C-terminal domain (CTD). These terminals are critical for V binding to DDB1, which is a cellular protein that is a component of the ubiquitin ligase E3 complex, as well as binding to MDA5 and LGP2, which are two host sensors of viral RNA. Here, we serendipitously discovered that the Hendra virus V protein undergoes a liquid-to-hydrogel phase transition and identified the V region responsible for this phenomenon. This region, referred to as PNT3 and encompassing residues 200–310, was further investigated using a combination of biophysical and structural approaches. Congo red binding assays, together with negative-staining transmisison electron microscopy (TEM) studies, show that PNT3 forms amyloid-like fibrils. Fibrillation abilities are dramatically reduced in a rationally designed PNT3 variant in which a stretch of three contiguous tyrosines, falling within an amyloidogenic motif, were replaced by three alanines. Worthy to note, Congo red staining experiments provided hints that these amyloid-like fibrils form not only in vitro but also in cellula after transfection or infection. The present results set the stage for further investigations aimed at assessing the functional role of phase separation and fibrillation by the *Henipavirus* V proteins.

## 1. Introduction

The Hendra and Nipah viruses (HeV and NiV) are members of the *Paramyxoviridae* family in the *Mononegavirales* order that is comprised of viruses with a non-segmented, single-stranded RNA genome of negative polarity. NiV and HeV are zoonotic agents responsible for severe encephalitis in humans that have been classified within the *Henipavirus* genus [1]. HeV emerged in 1994 in Brisbane, Australia, as a new pathogen responsible for an outbreak of an acute respiratory and neurological disease in horses. HeV still constitutes a threat to livestock in Australia, where sporadic and lethal transmission to humans has also occurred. NiV appeared in 1998 in Malaysia as the ethiologic agent of a respiratory and neurological disease mainly observed in pigs and humans. After the first cases of human infection in 1998 in Malaysia, NiV has regularly reemerged since 2001 in Bangladesh, India, and the Philippines with an average case fatality of 80%. The ability of NiV to be transmitted by direct inter-human transmission further extends its potential to cause deadly outbreaks [2,3,4]. The susceptibility of humans, their high pathogenicity, the wide host range and interspecies transmission, and the lack of vaccines and therapeutic treatments for humans led to the classification of henipaviruses as biosecurity level 4 (BSL-4) pathogens and as potential bio-terrorism agents. 

Like in all *Mononegavirales* members, the genome of henipaviruses is encapsidated by the nucleoprotein (N) within a helical nucleocapsid that serves as the substrate used by the viral polymerase for both transcription and replication. The viral polymerase is a complex made of the large (L) protein and the phosphoprotein (P). The P protein is an essential polymerase cofactor: not only does it recruit L onto the nucleocapsid template, but it also serves as a chaperon for it [5,6,7,8]. In addition, it chaperons the N protein, i.e., it prevents N self-assembly by binding to its monomeric form [9].

*Henipavirus* P proteins consist of an exceptionally long (>400 aa) N-terminal domain (NTD) that is intrinsically disordered [10,11] and a C-terminal region of approximately 300 aa containing two structured regions: a coiled-coil domain responsible for P multimerization (PMD) [12,13,14] and a triple α-helix X domain (XD) [15] that is responsible for the interaction with the C-terminal disordered domain (NTail) of the N protein [16,17,18,19]. 

**Figure 1:**
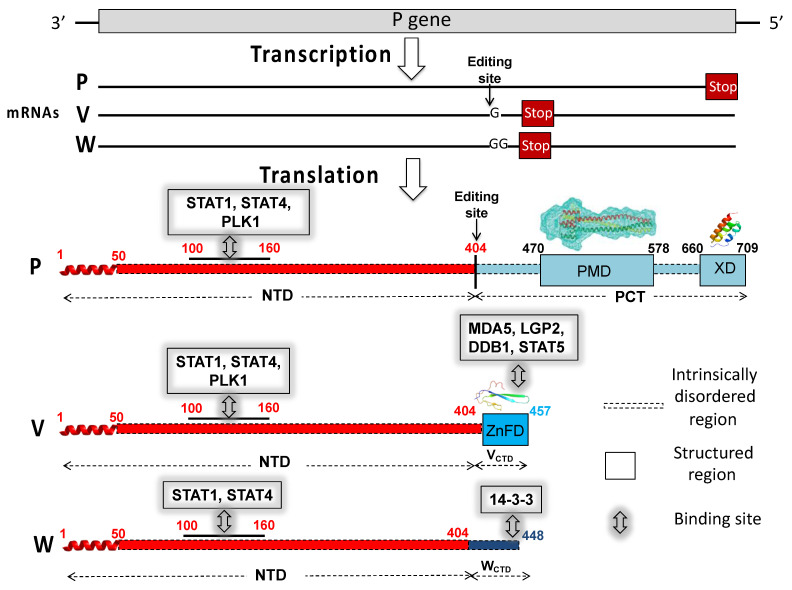
Coding capacity of the P gene and modular organization of the HeV P, V, and W proteins. Abbreviations: NTD, N-terminal region of P, V, and W; PCT, C-terminal region of P; PMD, P multimerization domain; XD, X domain of P; ZnFD, zinc finger domain; and V_CTD_ and W_CTD_, C-terminal domain of V and W. The α-helix at the N-terminus of P, V, and W corresponds to the region shown to adopt a stable α-helical conformation upon binding of P to N [9] or upon binding of V to host cellular transporters [33]. The crystal structure of HeV XD (PDB code 4HEO) [15] and a model of HeV PMD [13] are shown. The W_CTD_ is represented as disordered according to predictions [21]. Known interaction sites with human cell partners are shown.

In paramyxoviruses, the P gene also encodes the V and W proteins that are produced upon the addition of either one (protein V) or two (protein W) non-templated guanosines at the editing site of the P messenger. The addition of these guanosines triggers a frame shift downstream, resulting in a protein with a unique C-terminal domain (CTD). The editing site is located at the end of the region encoding the NTD. The P, V, and W proteins therefore share the NTD that can be considered as a bona fide domain (Figure 1). The CTD unique to paramyxoviral V proteins (V_CTD_) adopts a zinc finger conformation, with this folding being preserved both in isolation and in the context of the *Henipavirus* V proteins [20]. By contrast, the C-terminal domain unique to the *Henipavirus* W protein (W_CTD_) is predicted to be intrinsically disordered [21]. 

Paramyxoviral V and W proteins are key players in the evasion of type I interferon (IFN-I)-mediated responses [22,23,24]. *Henipavirus* V proteins prevent the detection of viral dsRNA by binding through their CTD to the RIG-like innate immune sensor melanoma differentiation-associated protein 5 (MDA5) and to the Laboratory of Genetics and Physiology 2 (LGP2) protein [25], which is an enhancer of dsRNA recognition by MDA5 [26]. *Henipavirus* V proteins also bind to PLK1 (polo-like kinase), another regulator of MDA5-dependent IFN-I induction, through their disordered NTD [27]. 

One of the key IFN signaling pathways relies on the activation of STAT (Signal Transducers and Activators of Transcription) proteins and the subsequent nuclear translocation. Once imported in the nucleus, STAT proteins interact with IRF-9 to form the ISGF3 complex that activates the transcription of IFN-stimulated genes (ISGs) whose products inhibit viral replication [22]. The V and W proteins of henipaviruses have an antagonist activity of IFN signaling [22,28]. They both bind to STAT1 via their NTD [29], leading to the inhibition of either STAT1 translocation into the nucleus (V) or STAT1 sequestration in the nucleus (W) [29]. The NTD region responsible for binding to STAT1 has been mapped to residues 114–140 [30], with this region having also been shown to bind to STAT4 [31]. Furthermore, the CTD of the NiV V protein binds to STAT5 [31]. In addition, *Henipavirus* V proteins interact via their CTD with the DNA damage-binding protein 1 (DDB1), a component of the ubiquitin ligase E3 complex [20]. By binding to both DDB1 and STAT proteins, *Henipavirus* V proteins promote rapid degradation of the latter. This ability to hijack the cellular ubiquitin ligase E3 complex is not unique to henipaviruses, as it has been already documented in several other paramyxoviruses [32]. 

Beyond their ability to antagonize IFN signaling, the V and W proteins also inhibit the production of chemokines in vitro and modulate the inflammatory response in vivo [34]. In addition, the *Henipavirus* W proteins bind to 14-3-3 proteins via their CTD, with this interaction resulting in the modulation of host gene exppression and apoptosis [35].

Although the *Henipavirus* P protein has an anti-IFN function as well, its IFN antagonist activity is moderate compared to V or W. This observation advocates for a critical role of the CTD of V and W in the anti-IFN function. In further support for a critical role of the CTD of V, in counteracting the host innate immune response, the NTD was shown to retain its overall disordered nature also in the context of the V protein [20], a finding that rules out the possibility that the NTD might adopt a unique conformation in the context of the V protein that could impart function to V. Rather, this argues for a scenario in which it is the C-terminal zinc finger domain (ZnFD) that specifically confers to the V protein the ability to counteract viral RNA recognition and IFN-I signaling. This function relies on the ability of the ZnFD to shield the RNA binding site of LGP2 and MDA5 [26], to enhance the binding to DDB1 [20], and to bind to STAT4 [31].

The pivotal role of *Henipavirus* V and W proteins in the evasion of the innate immune response is corroborated by the fact that the Cedar virus (the lastly discovered *Henipavirus* member), which lacks the V and W proteins, induces an IFN response much more pronounced compared to HeV, as well as induces an asymptomatic infection in animal models [36]. The fact that NiV and HeV are the paramyxoviruses with the highest frequency of the P messenger edition provides further support for the critical role of the V and W proteins in counteracting the innate immune response and hence in pathogenicity [23].

In the course of a further characterization of the *Henipavirus* V proteins, prompted by the fact that they are promising targets for antiviral approaches, we serendipitously discovered that the HeV V protein has the ability to form a hydrogel. In light of the growing number of studies pointing to a critical role of phase separations and transitions mediated by intrinsically disordered proteins (IDPs) and/or regions (IDRs) in various biological processes [37,38,39,40,41,42,43,44], we decided to investigate in detail this peculiar behavior.

Using a domain approach, we identified the V region responsible for this phenomenon and have further investigated it using a combination of biophysical and structural approaches. Using Congo red (CR) binding assays and negative-staining transmission electron microscopy (TEM), we show that this region forms amyloid-like fibrils. Through a mutational approach, we shed light on the sequence determinants underpinning this property. Finally, CR-staining of transfected and infected cells provided hints that fibrillation does not only occur in vitro but also in the cellular context. 

## 2. Materials and Methods

### 2.1. Generation of the Constructs 

The constructs encoding the HeV V (Uniprot code O55777) and NiV V (Uniprot code Q997F2) protein, as well as their NTD and ZnFD, have already been described [10,20].

The HeV PNT1, PNT2, PNT3, and PNT4 DNA fragments, encoding residues 1–110, 100–210, 200–310, and 300–404 of the HeV P/V protein, respectively, were PCR-amplified using the pDEST14/HeV PNT construct as a template [10] and as primers: namely PNT1-AttB1 and PNT1-AttB2; PNT2-AttB1 and PNT2-AttB2; PNT3-AttB1 and PNT3-AttB2; and PNT4-AttB1 and PNT4-AttB2, respectively. These primers were all designed to anneal on the desired region of the P gene, with forward primers starting with an AttB1 sequence and reverse primers ending with an AttB2 sequence. The resulting amplicons were then cloned into the pDest17 bacterial expression vector using the Gateway^®^ technology (ThermoFisher Scientific, France). This vector allows for the expression of the recombinant protein under the control of the T7 promoter. The resulting protein is preceded by a stretch of 22 vector-encoded residues (MSYYHHHHHHLESTSLYKKAGS) encompassing a hexahistidine tag.

For the prokaryotic expression of PNT3 fused to the green fluorescent protein (GFP), a 6His-tagged PNT3-GFP construct was generated by PCR (Polymerase Chain reaction) amplification using PNT3-pDest17 as a template and B1HisNT3 and NT3B2 as primers. After DpnI treatment, 1 µL of the first PCR was used as a template in a second PCR amplification using primers attl1a and attl2a as described in [45]. The second PCR product was then used in an LR reaction with the expression vector pTH31 [46].

For the eukaryotic expression of PNT3, a 6His-PNT3 construct was generated by PCR using His-PNT3-GFPq-pCDNA3.1+ (described in Supplementary Table S1) as a template and HindNT3 and NT3XhoI as primers. After DpnI treatment, the PCR product was digested by HindIII and XhoI, and ligated to pCDNA3.1+ as described above. 

For the prokaryotic expression of the PNT3 Y_211_A-Y_212_A-Y_213_A triple variant (PNT3^3A^), the His6-PNT3-pTH31construct was used as a template in two separate PCR amplifications using either primers attB1 and R_3ala-PNT3 (PCR1), or primers F_3ala-PNT3 and attB2 (PCR2). After DpnI treatment, 1 µL of PCR1 and 1 µL of PCR2 were used as overlapping megaprimers, along with primers attB1 and attB2 in a third PCR. After purification, the third PCR product was inserted into pDONR by BP reaction (ThermoFisher Scientific, Illkirch-Graffenstaden, France). After sequencing, the mutated coding sequence was transferred from pDONR to pDEST17 by LR reaction (ThermoFisher Scientific, Illkirch-Graffenstaden, France).

For the eukaryotic expression of the PNT3 Y_211_A-Y_212_A-Y_213_A triple variant (PNT3^3A^), the His-PNT3-pCDNA3.1+ construct was used as a template in two separate PCR amplifications using either primers HindNT3 and R_3ala-PNT3 (PCR1), or primers F_3ala-PNT3 and NT3XhoI (PCR2). After DpnI treatment, 1 µL of PCR1 and 1 µL of PCR2 were used as overlapping megaprimers, along with primers HindNT3 and NT3XhoI in a third PCR. After purification, the third PCR product was processed as described for generating His-PNT3-pCDNA3.1+. The list and sequence of the primers used to generate the above described constructs is provided in Supplementary Table S1.

The construct used for the prokaryotic expression of His-Hsp70 has already been described [47]. 

The constructs for the eukaryotic expression of the HeV F and G surface glycoproteins were obtained as follows. The genes encoding the full-length F protein and full-length of the G protein, deleted of the 32 residues from the cytoplasmic tail to ameliorate cell surface expression (referred to as G CT32), were cloned from the *wt* HeV in the pCAGGS plasmid. 

The construct driving the mammalian expression of the HeV V protein with a N-terminal flag tag (DYKDDDDK) was cloned from the virus cDNA. One G was introduced at the P editing site by PCR using primestar GXL polymerase (New England Biolabs, NEB, Évry-Courcouronnes France). The obtained amplicon was then introduced by In-Fusion (Takara) in the pcDNA3.1 plasmid, formerly digested with Bspe1 in 3.1 buffer (NEB) at 37 °C and purified after gel migration using nucleospin gel and the PCR clean-up minikit (Macherey-Nagel, Hoerdt, France).

Primers were purchased from Eurofins Genomics. All the constructs were verified by DNA sequencing (Eurofins Genomics, Ebersberg, Germany) and found to conform to expectations. 

### 2.2. Proteins Expression and Purification

The *E. coli* strain T7pRos transformed with one of the above described bacterial expression plasmids was used for the expression of all the recombinant proteins. Cultures were grown over-night to saturate in LB medium containing 100 µg mL^−1^ ampicillin and 34 µg mL^−1^ chloramphenicol. An aliquot of the overnight culture was diluted 1/20 into 1 L of TB medium and grown at 37 °C 200 rpm. When the optical density at 600 nm (OD_600_) reached 0.5–0.8, isopropyl β-d-thiogalactopyanoside (IPTG) was added to a final concentration of 0.5 mM and the cells were grown at 25 °C 200 rpm overnight. The induced cells were harvested, washed, and collected by centrifugation (5000 rpm, 10 min). The resulting pellets were frozen at −20 °C.

Expression of ^15^N-labelled PNT3 was performed as described above except that when the cultures reached an OD_600_ of 0.6, the culture was centrifuged at 4000 rpm for 10 min and the pellet was resuspended in 250 mL of M9 medium (6 g L^−1^ of Na_2_HPO_4_, 3 g L^−1^ of KH_2_PO_4_, 0.5 g L^−1^ of NaCl, and 0.246 g L^−1^ of MgSO_4_) supplemented with 1 g L^−1^ of ^15^NH_4_Cl and 4 g L^−1^ of glucose. After 1 h at 37 °C, IPTG was added to a final concentration of 0.5 mM and the cells were subsequently grown at 25 °C overnight.

The purification protocol of the *Henipavirus* V proteins and HeV V_CTD_ (i.e., ZnFD) have been already reported [20], as was that of HeV NTD [10]. The PNT1, PNT2, PNT3, and PNT4 proteins from pDEST17 constructs, and the PNT3-GFP protein from the pTH31 construct, were purified as follows. The frozen cellular pellet was thawed and resuspended (30 mL per liter of culture) in buffer A (50 mM Tris/HCl pH 7.5, 500 mM NaCl, and 20 mM imidazole) supplemented with 1 mM phenyl methyl sulfonyl fluoride (PMSF), 0.1 mg mL^−1^ lysozyme, 10 μg mL^−1^ DNAse I, and 20 mM MgSO_4_. After an incubation of 20 min at 4 °C, the cells were disrupted by sonication using a VCX750 sonicator (Sonics & Materials Inc., Newtown, CT, USA) and 3 cycles of 30 s each at 45% power output. The lysates were clarified by centrifugation at 14,000× *g* for 30 min at 4 °C and the supernatant was loaded onto a 5 mL Nickel column (GE Healthcare, Velizy Villacoublay, France) pre-equilibrated in buffer A. The affinity resin was washed with 20 column volumes (CV) of buffer A. Proteins were eluted with ~3 CV of buffer A supplemented with 250 mM imidazole. Eluted fractions were analyzed by SDS-PAGE, pooled and concentrated using Centricon concentrators (Merk-Millipore, Guyancourt France) (10 kDa molecular mass cut-off), and the proteins were further purified by size exclusion chromatography (SEC) using a Superdex S75 16/60 SEC column (GE Healthcare) and buffer B (20 mM Tris/HCl pH 7.5, 100 mM NaCl, and 5 mM EDTA) as the elution buffer. The fraction of interest were pooled, concentrated as described above, and stored at −20 °C.

In the case of PNT3 and PNT3^3A^, the purification protocol was subsequently modified as follows. Bacterial pellets were resupended in buffer A containing 6 M guanidium hydrochloride (GDN). After a short incubation with gentle agitation, the suspension was sonicated and then centrifuged as described above. The proteins were purified by immobilized metal affinity chromatography (IMAC) as described above. The fractions eluted from the Nickel column were pooled and concentrated in the presence of 6 M GDN up to 1–2 mM using Centricon concentrators, and the proteins were then frozen at −20 °C. Prior to each experiment, the PNT3 and PNT3^3A^ proteins were subjected to SEC that enabled the removal of GDN and allowed to assess the sample homogeneity along with the monomeric nature of the proteins. The SEC column was equilibrated with either buffer B (20 mM Tris/HCl pH 7.5, 100 mM NaCl, and 5 mM EDTA) or buffer C (50 mM sodium phosphate pH 6.5 and 5 mM EDTA). The latter was used for samples to be used in circular dichroism (CD), small-angle X-ray scattering (SAXS), TEM, and NMR experiments. In an alternative protocol, the fractions from SEC were pooled, supplemented with 6M GDN, and stored at −20 °C. Prior to each subsequent analysis, the sample was thawed and the buffer exchanged using Sephadex G-25 medium columns (GE Healthcare, Velizy Villacoublay, France). IMAC and SEC were performed at room temperature (RT).

Protein concentrations were estimated using the theoretical absorption coefficients at 280 nm as obtained using the program ProtParam from the EXPASY server. 

**Figure 2:**
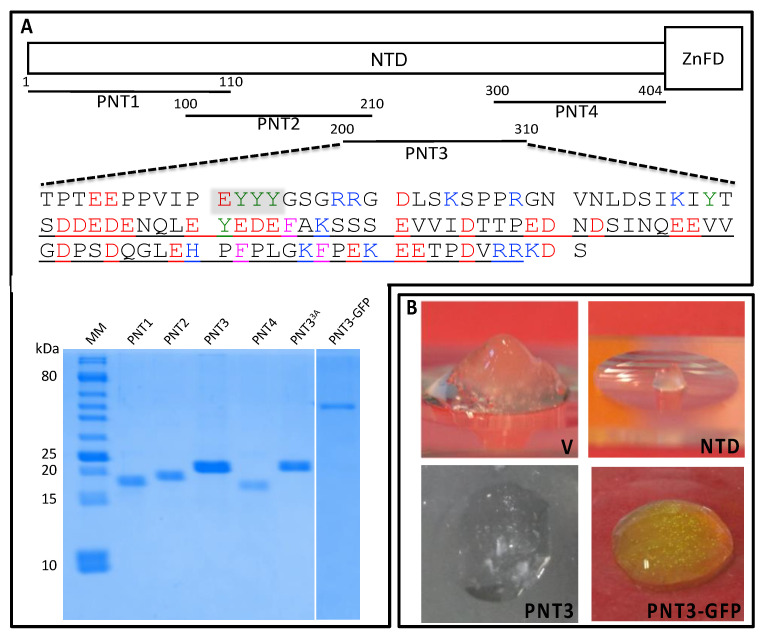
(**A**) Schematic organization of the HeV V protein (top) and SDS-PAGE analysis of purified proteins (bottom). The intrinsically disordered N-terminal domain (NTD) and the zinc finger domain (ZnFD) are represented as a narrow and large box, respectively. The various overlapping fragments within the NTD herein generated are shown. The amino acid sequence of PNT3 is shown, with the most amyloidogenic region, as predicted by FoldAmyloid [63], Aggrescan [64], and ArchCandy [65] (see Section 3.7.), framed in yellow. The low complexity region, as identified by SEG [66], is underlined. Tyr residues are shown in green, acidic residues in red, basic residues in blue, and phenylalanines in pink. SDS-PAGE analysis of the purified PNT1-4 fragments of PNT3^3A^ and of PNT3-GFP. Abbreviation: MM, molecular mass markers. (**B**) Hydrogels formed upon freezing and thawing of either purified V, NTD, PNT3 (all at 200 μM), or of purified PNT3-GFP (at 1 mM).

The purity of the final purified products was assessed by SDS-PAGE (Figure 2A). The identity of the purified PNT3 and PNT3^3A^ samples was confirmed by mass spectrometry analysis of tryptic fragments obtained after the digestion of the purified protein bands excised from SDS-polyacrylamide gels (Supplementary Figure S1). The excised bands were analyzed by the the mass spectrometry facility of Marseille Proteomics. Proteins were reduced by incubation with 100 mM dithiothreitol (DTT) for 45 min at 56 °C and free cysteine residues were capped by incubation with 100 mM iodoacetamide for 30 h at 25 °C in the dark. Samples were digested with porcine trypsin (V5111, Promega) at 12.5 ng/µL in 25 mM NH_4_HCO_3_ for 18 h at 37 °C. Peptides were extracted from the gel with 60% vol/vol acetonitrile in 5% formic acid, dried under vacuum, and reconstituted in 5 µL of 50% vol/vol acetonitrile in 0.3% trifluoroacetic acid. Mass analyses of the tryptic fragments were performed on a MALDI-TOF-TOF Bruker Ultraflex III spectrometer (Bruker Daltonics, Wissembourg, France) controlled by the Flexcontrol 3.0 package (Build 51) (Bruker Daltonics, Wissembourg, France). This instrument was used at a maximum accelerating potential of 25 kV and was operated in reflectron mode and an *m/z* range from 600 to 4500 (RP_Wide range_Method) or 600 to 3400 (RP_Proteomics_2015_Method). The laser frequency was fixed to 200 Hz and approximately 1000–1500 shots by sample were cumulated. Five external standards (Peptide Calibration Standard, Bruker Daltonics, Wissembourg, France) were used to calibrate each spectrum to a mass accuracy within 50 ppm. Peak-picking was performed with Flexanalysis 3.0 software (Bruker) with an adapted analysis method. Parameters used were as follows: the SNAP peak detection algorithm, the S/N threshold fixed to 6, and a quality factor threshold of 30. One µL of the sample was mixed with 1 µL of a saturated HCCA (α-cyano-4-hydroxycinnamic acid) solution in acetonitrile/0.3%TFA (1:1) and then 1 µL was spotted on the target, dried, and analyzed with the previously described method.

Hsp70 was purified according to [48] except that the last SEC step was replaced with a desalting step using a HiPrep 26/10 desalting column (GE Healthcare) and buffer B. The protein was then concentrated to 0.5 mM using Centricon concentrators (Merk-Millipore, Guyancourt France) with a 30 kDa molecular mass cut-off.

### 2.3. Turbidity Measurements 

Turbidity was measured by monitoring the optical density (OD) at either 600 or 340 nm using a NanoDrop ND-1000 (ThermoFisher Scientific, Illkirch-Graffenstaden, France) spectrophotometer. PNT3 samples (100 μL each), in the concentration range of 0–250 μM, were incubated for 1 h at RT either in the absence or in the presence of increasing concentrations of the crowding agent polyethylene glycol (PEG)_300_. Samples were also incubated for 1 h at RT or at 37 °C in the absence of PEG and in the presence of increasing concentrations of salt. 

### 2.4. Fluorescence Recovery after Photobleaching (FRAP) 

FRAP experiments were carried out on an Alexa-Fluor488 (FisherFisher Scientific, Illkirch-Graffenstaden, France)-labeled PNT3 sample. Labeling was performed by adding a 10-fold molar excess of Alexa Fluor488 to a PNT3 sample at 2 mg mL^−1^ in 50 mM sodium phosphate buffer, pH 7.5, 100 mM NaCl. After an incubation of 1 h at 37 °C, the excess dye was removed by gel filtration using a PD-10 (Sigma-Adrich, Saint-Quentin-Fallavier, France) column.

FRAP measurements were performed using an inverted Zeiss LSM 780 (Jena, Germany) with a Plan-Neofluar 40×/NA 1.4 DIC M27 oil immersion objective. All fluorescence images were collected at 0.5% with a 488 nm laser to prevent bleach acquisition as much as possible. The bleaching was performed by a single scan of the squared regions of interest of 4 µm^2^ inside PNT3-AF488 condensates with 100% of a 488 nm laser and a pixel dwell time of 65.54 µs. Each FRAP experiment contains 100 images: five pre-bleach and ninety-five post-bleach images. Experiments were conducted on different condensates to determine the standard deviation. 

Fluorescence recovery after bleaching curves were analyzed with a one phase exponential curve using Zen 2.3 SP1 FP3 black software (Carl Zeiss Microscopy GmbH, Jena, Germany).On each condensate, four regions were selected: two bleached regions, one region inside the condensate as a reference, and one region outside the condensate as a background reference. Raw data were normalized using Excel and plotted using GraphPad Prism 8.3.0 software (GraphPad Prism, San Diego, CA, USA). Fluorescence and bright-field images were formatted using ImageJ 1.53c software (http://imagej.nih.gov/ij).

### 2.5. Far-UV Circular Dichroism

CD spectra were measured using a Jasco 810 dichrograph (Jasco France, Lisses, France) flushed with N_2_ and equipped with a Peltier thermoregulation system. Proteins were loaded into a 1-mm quartz cuvette at 0.06 mg/mL (in 10 mM phosphate buffer at pH 6.5) and spectra were recorded at 37 °C. The scanning speed was 20 nm min^−1^ with a data pitch of 0.2 nm. Each spectrum is the average of five acquisitions. The spectrum of buffer was subtracted from the protein spectrum. Spectra were smoothed using the ‘‘means-movement’’ smoothing procedure implemented in the Spectra Manager package (Jasco France, Lisses, France). 

Mean molar ellipticity values per residue (MRE) were calculated as

(1) $$[\Theta ]=\frac{3300\;m\;\Delta A}{l\;c\;n}$$

where *l* is the path length in cm; *n* is the number of residues (133); *m* is the molecular mass in Daltons (15, 138); and *c* is the concentration of the protein in mg mL^−1^. 

### 2.6. Congo Red Binding and Shift Assays 

Congo red (CR, Sigma-Aldrich) binding assays were performed using the dye at a final concentration of 5 μM in the presence of 40 μM of either PNT3 or PNT3^3A^ in buffer C in a final volume of 50 μL. After a 3-weeks incubation at RT, the samples were centrifuged and the sedimented condensate was washed three times with 50 μL of buffer C. 

The quantitative measurement of CR binding (herein referred to as CR shift assay) was carried out using protein samples containing either PNT3 or PNT3^3A^ at 20 μM (in 50 mM sodium phosphate buffer at pH 7.2) in a final volume of 100 μL. The samples were incubated at 37 °C for 1 week and then CR was added to a final concentration of 5 μM. The adsorption spectrum of the CR solutions was recorded after 1 h of incubation at 37 °C using a PHERAstar FSX Microplate Reader (BMG LABTECH, Champigny-sur-Marne, France) in the 350–600 nm wavelength range. A solution of 5 μM CR in 50 mM sodium phosphate buffer with pH 7.2 without the protein was used as a control to normalize the analysis. A sample containing 5 μM of CR and 20 μM of the measles virus NTail protein, i.e., an IDP with no propensities to fibrillate [49], was used as a negative control. Statistical analysis was done using the one-way ANOVA test implemented in the PRISM 8.3.0. software (GraphPad Prism, San Diego, CA, USA). 

### 2.7. Small-Angle X-ray Scattering (SAXS)

SAXS and SEC-SAXS data were collected at the European Synchrotron Radiation Facility (ESRF, Grenoble, France) and at SOLEIL (Gif-sur-Yvette, France) as described in Table 1. In both cases, the calibration was made with water.

For SAXS studies, a PNT3 sample was subjected to SEC the day before using buffer C as an elution buffer. The fractions of interest were pooled and the sample was then kept in ice until data collection. Samples either at 1 or at 2 mg mL^−1^, as obtained upon dilution of the sample from SEC, were incubated at 37 °C and the data were collected at various times points (60, 90, 120, 180, 270, 300, 420, 480, and 630 min). Data reductions were performed using the established procedure available at BM29 and buffer background runs were subtracted from sample runs. 

For SEC-SAXS analyses, a lyophilized sample from a PNT3 solution at 3.5 mg mL^−1^ in buffer C was rehydrated and injected onto a BioSec 3-300 SEC column. Elution was carried out in the same buffer. Data reduction and frames subtraction were done with the beamline software FOXTROT (available upon request from the SOLEIL staff). Gaussian decomposition was performed using the UltraScan solution modeler (US-SOMO) HPLC-SAXS module (https://somo.aucsolutions.com/) [50] and the final deconvoluted scattering curves were submitted to the SHANUM program [51] to remove noisy, non-informative data at high angles.

For both types of experiments, data were analyzed using the ATSAS program package [51]. The radius of gyration (*R*_g_) and *I*(0) were estimated at low angles (*qR*_g_ < 1.3) according to the Guinier approximation [52,53]:

(2) $$Ln\left[I\left(q\right)\right]=Ln\left[I\left(0\right)\right]-\frac{q^{2}R_{g}^{2}}{3}$$

The pairwise distance distribution functions *P*(r) were calculated with the program GNOM [54]. The quality of the *P*(r) was always assessed with the CorMap test.

For the SAXS experiments, the *R*_g_ was calculated for both aggregates and monomeric forms whenever possible. To plot the normalized Kratky plot, the *R*_g_ and *I*(0) values of the monomer were used when clearly visible (60–180 min of incubation for the sample at 1 mg mL^−1^ and 60–90 min of incubation for the sample at 2 mg mL^−1^). The *R*_g_ and *I*(0) values of the major aggregated species were used for all the other incubation times.

Using the data collected at SOLEIL, we also attempted to describe PNT3 as a conformational ensemble. To this end, we used the program suite EOM 2.0 [55] using the sequence of the recombinant protein as input and default parameters (random coil conformers). The quality of the EOM fit was assessed with the CorMap test.

SEC-SAXS data of PNT3 at time-zero have been deposited in the Small Angle Scattering Biological Data Bank (SASBDB) [56] under code SASDLF9. The PNT3 ensemble derived using SEC-SAXS data at time-zero have been deposited within the Protein Ensemble Database (PED-DB, https://proteinensemble.org/) [57] under accession number PED00203.

The theoretical value of *R*_g_ (in Å) expected for an IDP was calculated using Flory’s equation according to [58]:

*R*_g_ = *R*_0_*N^ν^* (3)

where *N* is the number of amino acid residues, *R*_0_ is 2.54 ± 0.01, and *ν* is 0.522 ± 0.01.

### 2.8. Negative-Staining Transmission Electron Microscopy (TEM)

Drops of 2 μL of freshly purified PNT3 or PNT3^3A^ proteins (100–200 µM), either in the absence or in the presence of a two-fold molar excess of Hsp70, were deposited onto a glow-discharge carbon-coated grid (Formwar/Carbon 300 mesh Cu, Agar Scientific, Gometz la Ville, France). Prior to protein deposition, grids were exposed to plasma glow discharge for 20 s using a PELCO, easiGlow Glow Discharge Cleaning System (Ted Pella Inc. Redding, CA, USA) (current 25 mA), in order to increase protein adhesion. To assess fibrils stability, PNT3 (200 µM) supplemented with 5 mM SDS was deposited on the copper grids. The grids were washed three times with 20 µL of deionized water before incubating them for 1 min in 2% (*w/v*) Uranyl Acetate solution (Laurylab, Brindas, France). Images were collected using a TECNAI T12 Spirit microscope (FEI company, ThermoFisher, Illkirch-Graffenstaden France) operated at 120 kV and an Eagle 2Kx2K CCD camera (FEI company, ThermoFisher, Illkirch-Graffenstaden France).

### 2.9. Nuclear Magnetic Resonance (NMR)

A sample of a freshly purified ^15^N-labelled PNT3 at 100 µM in buffer C, also containing D_2_O (10%), was used for the acquisition of 1D ^1^H and 2D ^1^H,^15^N HSQC spectra with a 22.3 T Bruker AvanceIII 950 ultra-shielded-plus spectrometer equipped with a triple resonance CryoProbe (TCI) at 310 K (Bruker BioSpin GmbH, Rheinstetten, Germany). The sample was incubated at 37 °C and spectra were recorded at various time-points. Spectra were recorded both at 288 and 310 K. All the spectra were acquired, processed, and analyzed by using the Bruker software TopSpin 3.6.2 using standard parameters. Chemical shifts were referenced using DSS for ^1^H and indirect referencing for ^15^N using the conversion factor derived from the ratio of NMR frequencies [59].

### 2.10. SDS Sensitivity Assays

Preformed fibers of PNT3 (100 μM after 56 h of incubation at 37 °C in buffer C), either non-supplemented or supplemented with 2% (*w/v*) SDS, were passed through a 0.2 µm spin filter to remove fiber particles. The absorbance at 280 nm, after the subtraction of buffer contributions, was measured to monitor the amount of monomeric and small (i.e., ∅ < 200 nm) oligomeric protein species that passed through the filter.

In parallel, we also monitored the evolution of the aggregation index of a fibrillated PNT3 sample (180 μM after 72 h of incubation at 37 °C in buffer C) following incubation at RT either in the presence or in the absence of 2% SDS. To this end, the fibrillated PNT3 solution was diluted with buffer C to 110 μM and divided into two samples, of which one was supplemented with buffer C and the other with SDS to yield a final concentration of 2%. The final protein concentration was 100 μM. For each sample, 5 replicates were set up and the aggregation index was measured as a function of time.

The aggregation index was calculated as follows:

(4) $$\mathrm{Aggregation}\;\mathrm{Index}=\frac{OD340}{\left(OD280-OD340\right)}\times 100$$

Measurements were done on 100 μL samples using a PHERAstar FSX Microplate Reader (BMG Labtech, Champigny-sur-Marne, France) in the 220–600 nm wavelength range. The equality of variances between the different data sets was evaluated using Lavene’s test and Bartlett’s test. Statistical analysis was done using the one-way ANOVA test implemented in the PRISM 8.3.0 software (GraphPad Prism, San Diego, CA, USA).

### 2.11. Transfection of Mammalian Cells, CR-Staining, and Immunofluorescence Analysis

Human embryonic kidney (HEK) 293T cells were seeded in DMEM supplemented with 10% FBS at 37 °C in a Forma Series II Water Jacketed CO_2_ incubator (ThermoFisher Scientific, Illkirch-Graffenstaden, France). Transfections were performed 24 h after using the TransIT^®^-LT1 Transfection Reagent (Mirus) with a DNA:TransIT^®^-LT1 ratio of 1:3 (*w/v*).

Cells were seeded in Lab-Tek II chamber slides with covers (8 wells, 7 × 10^4^ cells/well) previously coated for 2 h at 37 °C with Poly-d-Lysin hydrobromide 50 µg/mL (Sigma-Aldrich) and were placed at 37 °C, 5% CO_2_. After 24 h, cells were transfected with 0.4 µg of either empty plasmids or plasmids encoding HeV V, or PNT3, or PNT3^3A^, or HeV F plus G CT32, and incubated at 37 °C, 5% CO_2_. The medium was then removed at various time intervals and cells were washed with phosphate-buffered saline (PBS, i.e., 137 mM NaCl, 2.7 mM KCl, 10 mM Na_2_HPO_4_, and 1.8 mM KH_2_PO_4_ pH 7.4). CR-staining experiments were performed 48 h after transfection. Cells were fixed with 4% PFA, washed with PBS, and incubated over night with 5 μM CR (Sigma-Aldrich) in 10 mM Hepes, 100 mM NaCl, pH 7.4. Cells were then washed for 1 h with PBS diluted in water (1/4) and stored at 4 °C in PBS before microscopy analysis.

Expression of PNT3, PNT3^3A^, and V in HEK 293T transfected cells was assessed 48 h post-transfection in Lab-Tek II chamber slides with covers (8 wells). Cells were washed with PBS, and fixed in methanol-free formaldehyde (Sigma Aldrich) 4% in PBS for 10 min at RT. After 3 washes in PBS, cells were then incubated in permeabilization and blocking buffer (PBB) containing PBS, 0.3% Triton X100, and 3% bovine serum albumine (BSA) for 20 min at RT. Media were then replaced with PBB containing the 6x-His Tag Monoclonal Antibody (HIS.H8, eBioscience™, Invitrogen) (at a 1:500 dilution) for 1 h at RT. After 3 washes, cells were incubated for 1 h at RT with the Donkey anti-Mouse IgG (H+L) Highly Cross-Adsorbed Secondary Antibody, Alexa Fluor 555, diluted 1:750 in PBB. Cells were washed in PBS and mounted in Fluoromount™ Aqueous Mounting Medium (Sigma Aldrich).

### 2.12. Infection and CR-Staining of Mammalian Cells

HEK 293T or HPMEC (human pulmonary microvascular endothelial) cells were seeded in µ-Slide 8 Well IBIDI Chambered Coverslip for Cell Imaging, previously coated for 2 h at 37 °C with Poly-d-Lysin hydrobromide at 50 µg/mL (Sigma-Aldrich) (7 × 10^4^ cells/well). The following day, cells were infected with HeV (Hendra virus/Australie/Horse/Hendra) (accession number: MN062017.1) at a multiplicity of infection (MOI) of 0.00025 for 1 h at 37 °C. Then, the medium was replaced with DMEM supplemented with 10% FBS (or endothelial cell growth medium with supplement from Promocell for HPMEC cells) and cells were replaced at 37 °C, 5%CO_2_, in humid atmosphere. After 48 h, cells were fixed with 4% PFA for one week. PFA (4%) was changed and cells were fixed for an additional week. Then, cells were washed in PBS and incubated overnight with 5 μM CR (Sigma-Aldrich) in 10 mM Hepes, 100 mM NaCl, pH 7.4. Cells were then washed for 1 h with PBS diluted in water (1/4) and stored at 4 °C in PBS before the microscopy analysis.

## 3. Results

### 3.1. Liquid-to-Hydrogel Transition by the HeV V Protein and Identification of the Region Responsible for This Behavior

In view of an in-depth structural characterization of the Henipavirus V proteins, we purified large amounts of both NiV and HeV V proteins at a relatively high concentrations (i.e., ≥10 mg/mL, 200 μM) and stored them at −20 °C in 20 mM Tris/HCl pH 8, 300 mM NaCl. Under these conditions, upon thawing the purified V protein samples, we noticed that HeV V, but not NiV V, formed a hydrogel (Figure 2B). This liquid-to-hydrogel transition is irreversible as neither dilution nor boiling can restore the liquid state, the latter being restored only upon addition of GDN at a final concentration of 6 M.

In order to identify the region responsible for this peculiar behavior, a domain approach was used. Out of the two domains of V (i.e., the NTD and the ZnFD), NTD was found to be sufficient to form a hydrogel under conditions similar to those under which HeV V jellifies (Figure 2B). The NTD was subsequently divided into four overlapping fragments (referred to as PNT1–PNT4) of 110 residues each (Figure 2A). The PNT fragments were all purified to homogeneity (Figure 2A). PNT3 (aa 200–310) was identified as the only fragment able to form a gel after a freezing/thawing cycle from a sample at 200 μM (Figure 2B). This ability is also retained in the context of a PNT3–GFP fusion, although gel formation was observed at a higher (i.e., 1 mM) protein concentration (Figure 2B).

Bioinformatics analysis carried out using the phase-separation predictors PSPredictor (http://www.pkumdl.cn:8000/PSPredictor/) [60], catGranule (http://s.tartaglialab.com/update_submission/365826/b2ed515dd0) [61], and FuzPred (http://fuzpred.med.unideb.hu/fuzpred/upload_fasta.php) [62] all identified the HeV V protein and its NTD as capable of undergoing phase separation (Supplementary Table S2). In accordance with the experimentally observed inability of the NiV V protein to form a gel upon a freezing/thawing cycle under conditions in which the HeV V protein does so, all the above-listed predictors consistently returned a lower phase-separation score for NiV V compared to HeV V (Supplementary Table S2).

Surprisingly, when the PNT3 sequence was analyzed on its own, only FuzPred predicted it as a sequence with a significant LLPS propensity (see Supplementary Table S2). In spite of the fact that two out of three predictors do not identify PNT3 as a phase-separating protein, its ability to jellify can be rationalized in light of bioinformatics analyses that unveiled the presence of a low complexity region (enriched in Glu) encompassing residues 240–307 (Figure 2A). Low complexity domains are indeed known to drive the physiologically reversible assembly of IDPs into membrane-free organelles, liquid droplets, and hydrogel-like structures [41,62,67,68,69,70]. In addition, PNT3 contains a stretch of three contiguous tyrosines (aa 211–213 of V) and two non-contiguous tyrosines at positions 238 and 250 (Figure 2A). The well-established effect of tyrosines [71] and, more generally, of π-orbital containing residues [72,73,74] in promoting phase separation provides an additional conceptual piece to rationalize the behavior of PNT3.

### 3.2. Phase-Separation Abilities of PNT3

Protein-containing gels can result from the maturation of liquid condensates deriving from liquid–liquid phase separation (LLPS) [38,41,75]. We thus first assessed whether PNT3 phase separates and then investigated the conditions under which this phenomenon occurs.

**Figure 3:**
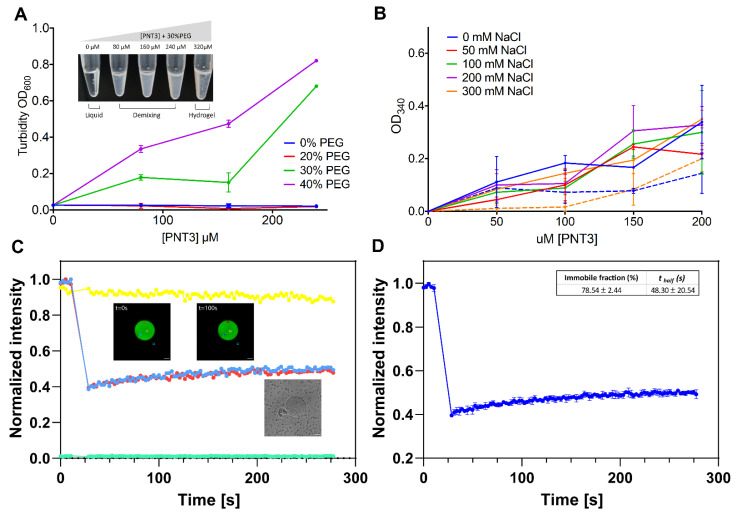
Phase-separation abilities of PNT3. (**A**) Turbidity measurements of PNT3 samples at different concentrations either in the absence or in the presence of increasing PEG_300_ concentrations after 1 h of incubation at RT. (**B**) Aggregate formation in PNT3 samples at different concentrations either in the absence or in the presence of increasing concentrations of NaCl after 1 h of incubation at 37 °C (continuous lines) or at RT (dotted lines). (**C**,**D**) present the FRAP analysis of an Alexa-Fluor 488-labeled PNT3 sample at 100 nM after a one-week incubation at 37 °C. (**C**) Fluorescence recovery curves of two condensate (blue and red) and two control (yellow and green) regions. Insets: fluorescence images of condensate at *t* = 0 s and *t* = 100 s, and bright-field image of the condensate. Scale bars: 10 µm. (**D**) Fluorescence recovery curve of PNT3-AF488 condensates. Typical error bars represent the standard deviation (SD) of measurements on four different regions of the condensate, including the blue and red regions shown in panel C.

After 1 h of incubation at RT in the presence of a crowding agent (i.e., 30% PEG_300_), PNT3 was found to phase separate in the 80–240 μM range and to form a hydrogel at 320 μM (see inset in Figure 3A). Phase separation can be quantified by turbidity measurements (Figure 3A) that show that the phenomenon is dependent on both protein and PEG concentration, with a PEG concentration of 20% having no significant impact. We next assessed whether PNT3 retains the ability to phase separate in the absence of a crowding agent and investigated the impact of salt and temperature. To this end, turbidity measurements were carried out at 340 nm, a wavelength that is more sensitive to protein aggregation than 600 nm [76]. As shown in Figure 3B, the turbidity increases in a concentration-dependent manner even in the absence of PEG. Interestingly, salt does not seem to significantly affect the ability of the protein to form condensates, at least in the concentration range herein explored, suggesting that the phenomenon relies on hydrophobic rather than electrostatic interactions. The formation of phase-separated condensates was slightly enhanced at 37 °C (Figure 3B).

Turbidity measurements cannot discriminate between liquid and solid condensates. In order to assess the nature of the PNT3 condensates, we performed FRAP experiments (Figure 3C,D and Supplementary Movies S1 and S2). From the evolution of the fluorescence intensity within the region of interest (Figure 3C), the rate of (or half-time for) fluorescence recovery of a photobleached component and the extent of fluorescence recovery (referred to as the mobile fraction) can be derived. Fast exchange rates (in the second range) and high percentages (~80%) of mobile fraction characterize liquid-like assemblies. The immobile fraction value observed (78.54 ± 2.44%) is consistent with a solid-like state (Figure 3D). The low amplitude of the error bars obtained from measurements on four different condensate regions (Figure 3D) indicates that the condensate was spatially homogeneous in terms of its material properties. These experiments, beyond shedding light onto the nature of the PNT3 condensates, also confirmed the ability of PNT3 to phase separate in the absence of crowding agents and revealed that the process takes place even in the sub-micromolar concentration range, although a prolonged incubation period at 37 °C was required. However, neither turbidity nor FRAP measurements enable the distinguishing between amorphous and non-amorphous aggregates.

### 3.3. Fibrillation Abilities of PNT3

Taking into account that solid-like condensates resulting from phase separation can nucleate amyloid-like fibrils [38,75], we analyzed the ability of PNT3 to bind the amyloid-specific dye CR [77]. Binding of CR to cross β-sheet structures is known to lead to hyperchromicity and a red shift of the absorbance maximum [77].

**Figure 4:**
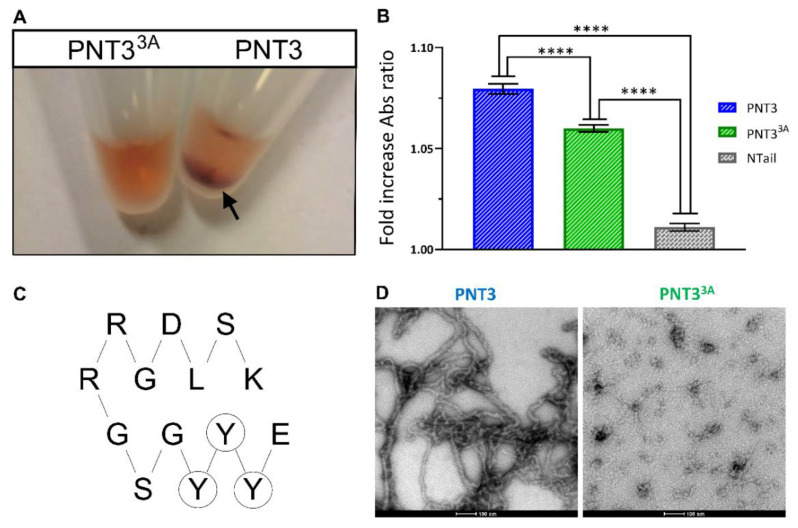
(**A**) CR binding by purified PNT3 and PNT3^3A^. Proteins were incubated at 40 μM in the presence of 5 μM CR for 3 weeks at RT in 50 mM sodium phosphate pH 6.5, 5 mM EDTA. The arrow points to the dense phase on the bottom of the tube that binds the CR observed for PNT3 but not for PNT3^3A^. (**B**) Fold increase in the ratio between the absorbance at 515 and the absorbance at 497 nm, with respect to a sample containing CR alone (at 5 μM), of a PNT3, or PNT3^3A^, or NTail (control) sample at 20 μM after 1 h of incubation at 37 °C. Note that PNT3 and PNT3^3A^ were incubated for 1 week at 37 °C before the addition of CR. The error bar corresponds to the standard deviation, with *n* = 9 for PNT3 and PNT3^3A^, and *n* = 5 for Ntail. The four asterisks denote a statistically significant difference (*p* < 0.0001) (one-way ANOVA test). (**C**) Output was provided by ArchCandy [65] when submitting the amino acid sequence of PNT3. The three contiguous tyrosines within the amyloidogenic EYYY motif, which were targeted for mutagenesis, are circled. (**D**) TEM micrographs of a PNT3 or PNT3^3A^ sample at 200 μM after a 56-h incubation at 37 °C.

PNT3 was indeed found to form macroscopically observable condensates that sediment on the bottom of Eppendorf tubes and that bind CR (see arrow in Figure 4A). To quantify this phenomenon, the red shift of the absorbance maximum in the CR spectrum of a sample containing PNT3 was spectrophotometrically measured. The addition of PNT3 does indeed promote a significant, though moderate, shift in the CR spectrum from 497 nm to 515 nm (Figure 4B). These observations provide the first hints suggesting that PNT3 can form β-enriched/amyloid-like structures. Interestingly and surprisingly, PNT3 was found to be unable to enhance the fluorescence intensity of Thioflavin T, another amyloid-specific dye [78], in a robust and reproducible manner.

**Figure 5:**
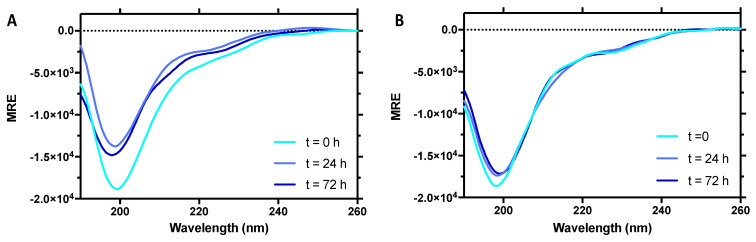
Far-UV circular dichroism (CD) studies of PNT3 (**A**) and PNT3^3A^ (**B**). The spectra were recorded in 10 mM sodium phosphate pH 6.5 at 37 °C. Protein concentration was at 0.06 mg mL^−1^ (4 μM). The spectra shown in cyan correspond to a freshly purified PNT3 or PNT3^3A^ sample recorded immediately after elution from the SEC column. The spectra shown in light and dark blue were recorded from the same samples incubated at 37 °C for 24 h and 72 h, respectively. Note the much more pronounced decrease in the signal for the spectrum at 24 h of PNT3 compared to PNT3^3A^. MRE (Θ) is expressed in deg cm^2^ dmol^−1^.

To achieve additional insights on the secondary structure content of PNT3, we recorded the far-UV CD spectra of PNT3 as a function of time (Figure 5). The CD spectrum of a freshly purified sample of PNT3 was typical of an IDP, as illustrated by the very pronounced negative peak at 200 nm and low ellipticity in the 190–200 nm region (Figure 5). After a 24-h incubation at 37 °C, a dramatic decrease in the signal was observed with no concomitant change in the spectral shape. This phenomenon likely arises from the formation of fibrils that cannot be crossed by the polarized light and was also observed in the case of phase-separated Pro-Arg dipeptide repeats [69]. Note that the possibility that the loss of signal might arise from protein degradation was checked and ruled out by SDS-PAGE (data not shown). After an additional 48-h incubation, only a slight decrease in the overall spectral intensity was observed, suggesting that the events that concurred to reduce the signal mostly took place during the first 24 h. The overall shape of the spectra does not change, which suggests that the species that contributes to the signal maintains its prevalently disordered nature. By contrast, the possibility that structural transitions can occur within the fibrillar species escaping detection by CD cannot be ruled out.

Negative staining transmission electron microscopy (TEM) unequivocally confirmed the presence of amyloid-like fibrils (Figure 4D). Altogether, these data provide strong evidence for the presence of amyloid-like polymers as the structural basis of phase-separated solid-like PNT3 condensates. Notably, fibril formation does not require a liquid-to-hydrogel transition, indicating that phase-separated condensates nucleate amyloid-like fibers in the liquid state. Considering gelation occurs upon a freezing/thawing cycle of a freshly purified sample in the pre-fibrillation state, it seems that the fibrillation process is not a prerequisite for gelation.

### 3.4. Small-Angle X-ray Scattering Studies of PNT3

**Figure 6:**
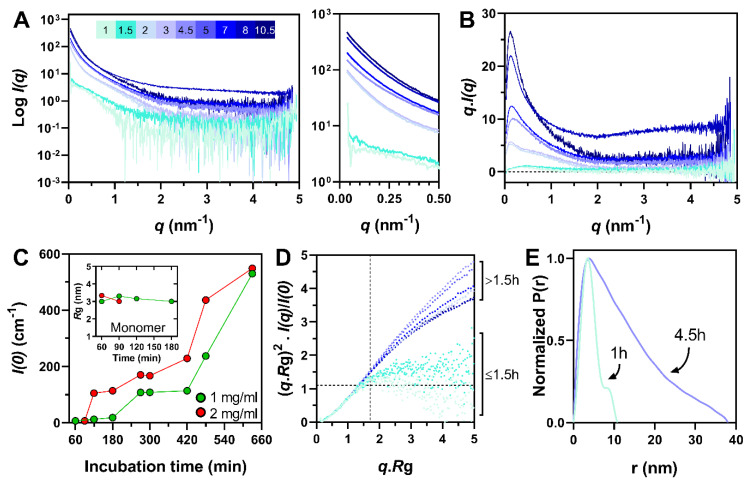
PNT3 aggregation process followed by SAXS. (**A**) Scattering intensities of PNT3 at 2 mg mL^−1^ (132 μM), recorded from 1 h to 10.5 h of incubation at 37 °C. A zoomed-in view of the low-angle region is shown on the right. (**B**) Total scattering intensities. The color code is the same as in (**A**). (**C**) Zero-angle scattering intensities (*I*(0)) as a function of the incubation time. Insert: estimated *R*_g_ for the monomeric species. (**D**) Normalized Krakty plot. The black dotted lines indicate the maximum of a bell-shaped curve as observed for globular proteins. The color code is the same as in (**A**,**B**,**E**) Normalized pairwise distance distribution functions of PNT3 incubated for 1 h and 4.5 h.

In view of achieving a better description of the evolution of the conformational properties of PNT3 over time, we carried out SAXS studies. Synchrotron SAXS data were collected from a PNT3 sample at two different concentrations (1 and 2 mg mL^−1^) and at different times of incubation (from 1 h to 10.5 h) at 37 °C (Figure 6 and Supplementary Figure S2). At 2 mg mL^−1^ (132 μM), the calculated radii of gyration (*R*_g_) obtained after 1 h (*R*_g_ = 3.34 ± 0.1 nm) and 1.5 h (*R*_g_ = 3.01 ± 0.08 nm) of incubation are consistent with the value expected for a monomeric form of PNT3 in a disordered state according to Flory’s equation (*R*_g_ = 3.26 ± 0.03 nm) [58]. After 2 h of incubation, higher order oligomers/aggregates dominated, as judged from the intensity increase in the low-angle region of the scattering curves (Figure 6A) and from the shape of the total scattered intensities plots (Figure 6B). By contrast, at 1 mg mL^−1^ (66 μM), higher order oligomers/aggregates were detected only after 4.5 h (Supplementary Figure S2A,B). Indeed, at this concentration, the monomeric form persisted for up to 3 h of incubation according to the calculated *R*_g_ (Figure 6C, inset). In agreement, the forward scattering intensity at zero angle *I*(0), which is proportional to the mass of the scatterer, increased faster and to a larger extent for the sample at 2 mg mL^−1^ (Figure 6C). The kinetics of the formation of oligomers and/or fibrils was thus accelerated at a higher protein concentration.

For the samples containing higher order oligomers/aggregates and suffering from high polydispersity, the *R*_g_ were calculated at the lowest angles so as to characterize the biggest species and the derived values (see Material and Methods section) were used to draw the normalized Kratky plots (Figure 6D and Supplementary Figure S2C). Surprisingly, at the higher protein concentration, the PNT3 sample incubated for 1 h at 37 °C seems to be almost globular, as judged from the bell shape of the plot (Figure 6D), a finding in contrast with the calculated *R*_g_ that is close to the value expected for an IDP (Figure 6C, inset). By contrast, after 1.5 h of incubation, PNT3 appeared, as expected, to be disordered, as inferred from the presence of a plateau in the normalized Kratky plot (Figure 6D). At the lower protein concentration, the protein remained disordered for up to 2 h and then appeared to be almost globular after 3 h of incubation (Supplementary Figure S2C). Again, the bell-shaped curve observed after 3 h of incubation is in contrast with the calculated *R*_g_ value (Figure 6C, inset) that reflects a disordered state. This puzzling behavior may have arisen from the poor signal-to-noise ratio arising in its turn from the polydispersity of the samples.

The curves obtained for the sample at 2 mg mL^−1^ incubated for 2 h and up to 4.5 h progressively lost their initial shape, becoming more and more linear in the studied q.*R*g range (Figure 6D), consistent with the formation of extended particles with a rod-like shape [79]. This observation is supported by the shape of the pairwise distance distribution function after 4.5 h of incubation that exhibited features typical of rod-like particles (Figure 6E). This behavior is consistent with the formation of fibrillar species [80]. As expected, the formation of elongated structures was delayed at 1 mg mL^−1^ and appeared only after 4.5 h of incubation (Supplementary Figure S2C).

**Figure 7:**
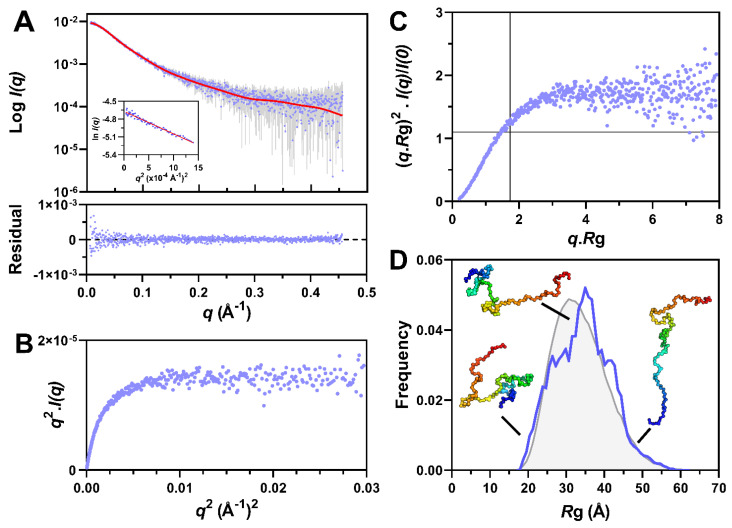
SEC-SAXS analysis of the monomeric form of PNT3. (**A**) Scattering intensities of PNT3 as resulting from the Gaussian deconvolution (blue) and EOM fit (red line). The bottom panel shows the plot of residuals between the deconvoluted scattering curve and the curve back-calculated from the EOM ensemble. Insert: *R*_g_ determination according to the Guinier approximation. (**B**) Kratky–Debye plot. (**C**) Normalized Kratky plot. Black lines indicate the maximum of a bell-shaped curve observed for globular proteins. (**D**) *R*_g_ distribution of the ensemble selected by EOM (blue, average *R*_g_ = ~35 Å) and of the initial random coil pool of conformers (grey, average *R*_g_ = ~34 Å). Three representative low-resolution conformers are shown. The structures were drawn using Pymol 2.0.1 (https://pymol.org/2/) [82].

Finally, in order to obtain an ensemble description of the monomeric species, we sought to use the program suite EOM 2.0 [55]. To this end, we used data collected by SEC-SAXS so as to ensure the maximal monodispersity of the sample. In addition, data were collected both without any prior incubation at 37 °C, so as to enable analyzing the sample at time zero, and after 1 h of incubation. In both cases, the SEC elution profile features a major peak corresponding to the monomeric species and an additional peak corresponding to aggregates (Supplementary Figure S3A). The peak of the monomeric form is not perfectly symmetric and displays a shoulder at ~10 mL (Supplementary Figure S3A). In line with this, the *I*(0) and *R*_g_ plot indicates the presence of a low-abundance high-molecular mass contaminant (Supplementary Figure S3B and data not shown). In order to remove the contribution of this species to the scattering data, Gaussian decompositions were performed using US-SOMO [50]. The resulting scattering curves display linearity in the Guinier region (Figure 7A and Supplementary Figure S3C), thereby allowing for a meaningful estimation of the *R*_g_.

The *R*_g_ of PNT3, as derived from the Guinier plot (Figure 7A, inset), is 3.35 ± 0.03 nm, a value nearly identical to that obtained from the in-batch SAXS studies for the sample at 2 mg mL^−1^ after 1 h of incubation (*R*_g_ = 3.34 ± 0.1 nm). Incubation of the sample for 1 h at 37 °C led to the formation of a higher amount of aggregates but did not cause any variation of the scattering profile of the monomeric species as shown by the CorMap *p*-value (0.111) (Supplementary Figure S3A,C). The latter is a measure of goodness-of-fit that estimates the differences between one-dimensional spectra, independently of explicit error estimates, using only data point correlations [81].

At time zero, PNT3 was disordered as judged from the presence of a plateau in both the Kratky–Debye (Figure 7B) and normalized Kratky (Figure 7C) plots. This conclusion holds true also after 1 h of incubation, as the corresponding scattering curve is superimposable to the one obtained without any prior incubation (Supplementary Figure S3C).

The scattering curve of the monomeric form of PNT3 as obtained at time zero was thus used as input for EOM (Figure 7D). From an initial pool of 10,000 random-coil conformations, EOM selects a sub-ensemble of conformers that collectively reproduces the experimental SAXS data and represents the distribution of structures adopted by the protein in solution. The average SAXS scattering curve back-calculated from the selected sub-ensemble correctly reproduces the experimental curve (Figure 7A) as shown by the plot of residuals (Figure 7A, bottom panel) and by the CorMap p-value (0.62). The final ensemble consists of eight conformers (of which six are unique).

The *R*_g_ distribution of the selected sub-ensemble, as obtained from two independent EOM runs, is almost unimodal, centered on ~35 Å, and similar to that of the initial pool of random-coil conformers (Figure 7D). The similarity of the *R*_g_ distributions obtained in different EOM runs attests to the reproducibility of the selection process and hence the reliability of the inferred conformational information.

The distribution of the maximum particle sizes (D_max_) of the selected ensemble ranges from ~50 to 200 Å, centered on ~100 Å (Figure S3D). The selected ensemble exhibits a high flexibility (R_flex_ = 88.4 %), a value similar to that of the initial pool (87.2%, Rσ = 1.04) and consistent with pure random-coil conformations.

In conclusion, SAXS experiments show that PNT3 was monomeric and disordered in the solution, adopting a typical Gaussian chain distribution of its parameters. However, it rapidly aggregates in a concentration-dependent manner to form rod-like particles, a behavior compatible with the formation of fibrillar species. Moreover, these experiments did not enable the capturing of any transition state between the monomeric and fibrillar form of PNT3, indicating rapid aggregation kinetics.

### 3.5. Nuclear Magnetic Resonance (NMR) and Negative-Staining Transmission Electron Microscopy (TEM) Studies of PNT3

We next carried out negative-staining TEM and heteronuclear NMR studies to directly document fibril formation as a function of time and to reveal any possible concomitant structural transition.

**Figure 8:**
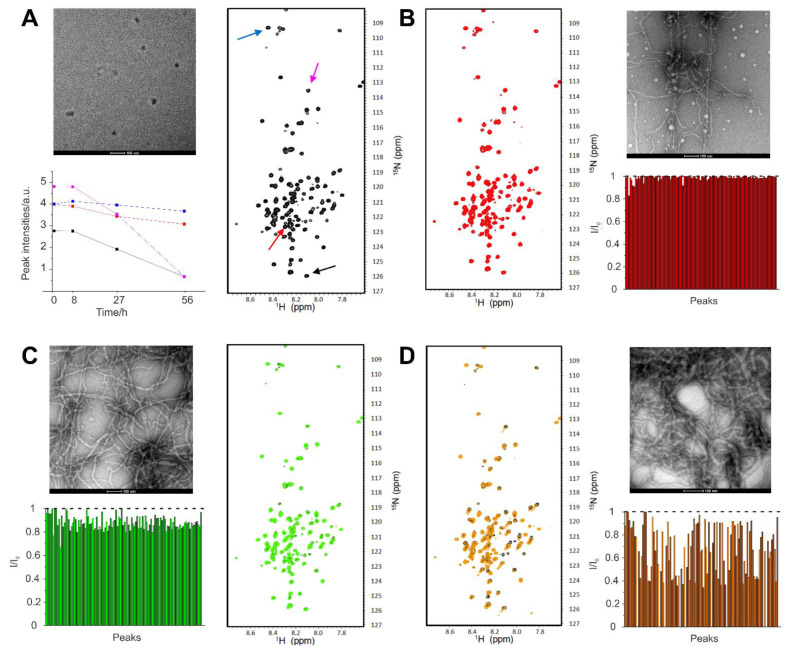
^1^H-^15^N HSQC spectra of a 100 μM PNT3 sample in 50 mM phosphate buffer at pH 6.5, 5 mM EDTA after 0 (**A,** black), 8 (**B,** red), 27 (**C**, green), and 56 (**D**, orange) h of incubation at 37 °C. The HSQC spectrum of the sample at time 0 is shown in black in all panels. The peak intensities ratio between the amide protons signals of the sample recorded after 8 (**B**), 27 (**C**), and 56 (**D**) h of incubation at 37 °C, and those of the sample at time 0, are shown on the sides of panels B, C, and D, respectively. The intensity variation of four representative peaks is shown in the plot present in panel A and the associated peaks are indicated by arrows in the spectrum. The spectra were recorded at 310 K. Insets: negative-staining TEM micrographs of a PNT3 sample at 200 μM incubated at 37 °C for 0, 8, 27, and 56 h in the same buffer.

We thus recorded the ^1^H-^15^N HSQC spectra of a uniformly labeled PNT3 sample after various incubation times at 37 °C. As shown in Figure 8, a time-course analysis of the sample did not reveal any significant chemical shift variations of the signals. Rather, an overall progressive reduction in cross-peak intensities was observed with increasing incubation time. The observed reduction in peak intensities is reminiscent of that observed in CD studies (see Figure 5) and is likely attributable to the formation of fibrillar species that no longer contribute to the solution-state NMR signals, as already observed in the case of phase-separated droplets containing the N and P proteins from measles virus [83]. In the spectrum recorded after 56 h of incubation, some secondary signals also appeared, which are likely associated to the presence of multiple phases. Interestingly, the intensity variation was not uniform for all the signals and is likely more significant for the residues involved in the formation of the core of the fibrils, whose identification requires and awaits assignment of the PNT3 resonances.

A concomitant analysis with negative staining TEM unambiguously showed the progressive formation of amyloid-like fibrils. While those fibrils became progressively more abundant over time, their diameter (12–17 nm) seemingly remained unvaried.

### 3.6. Impact of SDS and Heat-Shock Protein 70 (Hsp70) on PNT3 Fibrils

Extreme stability is a hallmark of pathogenic amyloid fibers (see [84,85] and references therein cited). In addition, yeast ultra-stable amyloids, derived from the low complexity sequences associated with transcription factors and RNA-binding proteins, have also been described [86]. These prion-like amyloid fibers share a common insensitivity to the solubilizing effects of SDS.

**Figure 9:**
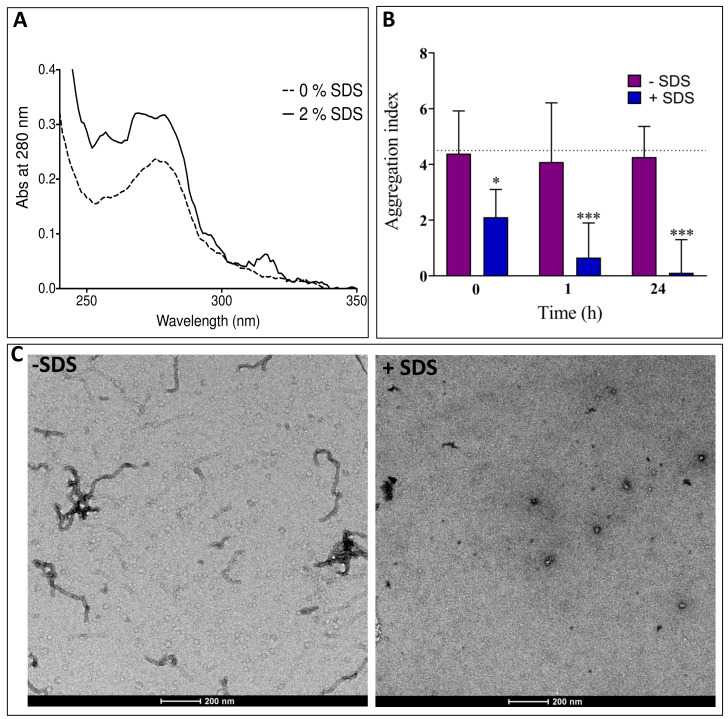
(**A**) Absorbance at 280 nm of the filtrate of a sample of PNT3 at 100 μM after 56 h of incubation at 37 °C before (dashed line) and after (continuous line) exposure to 2% SDS, and 10 min incubation at 37 °C. Exposure of SDS enables more material to pass through the filter. (**B**) Aggregation index of a fibrillated sample of PNT3 at 100 μM before and after incubation at RT either in the presence or in the absence of 2% SDS. Exposure to SDS reduces the aggregation index. The error bar corresponds to the standard deviation with *n* = 5. The asterisks denote a statistically significant difference with respect to the sample without SDS (* *p* < 0.0265; *** *p* < 0.0004, one-way ANOVA test). (**C**) Micrographs of a PNT3 sample at 200 μM after 56 h of incubation at 37 °C either in the absence or in the presence of 5 mM (1.44 mg mL^−1^) SDS.

In order to investigate the SDS sensitivity of PNT3 fibers, heavily polymerized preparations of PNT3 (i.e., 100 μM after 56 h of incubation at 37 °C) were filtrated through a membrane allowing for the passage of only monomeric and oligomeric forms with a diameter of less than 200 nm. As shown in Figure 9A, a very small amount of protein was found to pass through the filter when the fibers were diluted in standard buffer and filtrated immediately. In contrast, following incubation in the presence of 2% SDS at 37 °C for 10 min, the amount of UV-adsorbing material passing into the filtrate increased. These results suggest that the fibers are at least partly depolymerized into monomers and smaller oligomers by SDS treatment.

In a similar manner, the incubation of a fibrillated PNT3 sample (as obtained after incubation for 72 h at 37 °C) in the presence of 2% SDS led to a decrease in the aggregation index compared to a sample incubated in the absence of SDS (Figure 9B). These results therefore confirm that SDS is capable of at least partly disassembling aggregated species, with this effect being discernible immediately after the addition of the detergent (Figure 9B). The disassembly effects were further increased after 1 h of incubation and even more pronounced after 24 h of incubation (Figure 9B). A time-course analysis as a function of hours showed that the disassembly effect reached a plateau as soon as after 2 h (Supplementary Figure S4). The ability of SDS to depolymerize PNT3 fibers is further supported by TEM studies that revealed that in the presence of 5 mM, SDS fibers disappeared (Figure 9C). Indeed, the analysis of up to ten grid squares revealed the presence of as few as two small fibers all over. Thus, in the presence of SDS, PNT3 fibrils showed a low stability, in line with previous observations on stress granule proteins [87] and on α-synuclein, tau, and Aβ42 fibrils [88].

Therefore, although PNT3 fibers share morphological similarities with the prion-like fibers broadly described in the literature, they appear to be more fragile.

**Figure 10:**
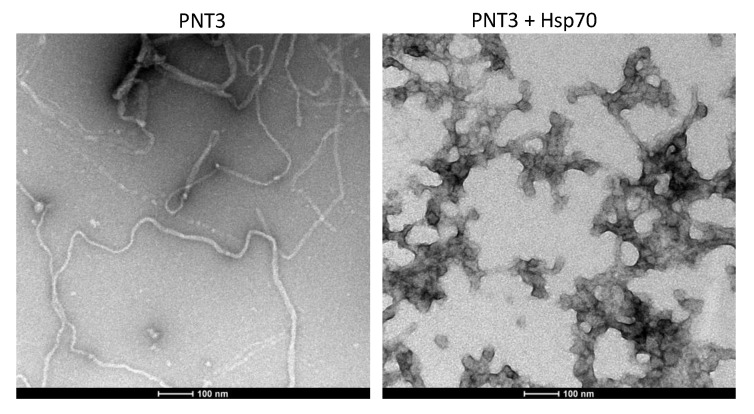
TEM micrographs of a PNT3 sample at 100 μM incubated at 37 °C for 27 h either in the absence or in the presence of a two-fold molar excess of Hsp70.

In light of previous studies that documented the ability of chaperons and, in particular, of the major inducible heat shock protein 70 (Hsp70) to inhibit or delay fibril formation by prion-like proteins [89,90,91,92,93,94], we sought at ascertaining whether human Hsp70 has an impact on the fibrillation process of PNT3. In line with expectations, TEM studies showed that in the presence of Hsp70, the formation of fibrils was hampered (Figure 10). Specifically, the addition of hsp70 leads to the disappearance of fibrillar structures in favor of amorphous assemblies (Figure 10), a scenario already observed in the case of α-synuclein and as ascribed to the formation of the amorphous aggregates of both α-synuclein and Hsp70 [90].

### 3.7. Rational Design of a PNT3 Variant with a Hampered Ability to Form Amyloid-like Fibrils

With the goal of generating a rationally designed, non-amyloidogenic variant of PNT3, we carried out a bioinformatic analysis. Taking into account the ability of prion-like domains (PLDs) (i.e., IDRs enriched in Asn and Gln residues) to drive fibrillation [38,41,95,96,97,98], we first analyzed the PNT3 sequence using various PLD predictors including LPS, PAPA, PLAAC, and PrionW (see [99] and references therein cited). No PLD was found within the PNT3 sequence, indicating that the sequence determinants that drive the fibrillation of PNT3 are distinct from those of typical PLDs. In search of amyloidogenic regions, we also used several other computational methods. As a result, we identified the EYYY motif (aa 210–213) as the common amyloidogenic region consistently predicted by three predictors. In fact, FoldAmyloid (http://bioinfo.protres.ru/fold-amyloid/) [63], Aggrescan (http://bioinf.uab.es/aggrescan/) [64], and ArchCandy (https://bioinfo.crbm.cnrs.fr/index.php?route=tools&tool=7) [65] predicted P**EYYY**, **EYYY**G, and **EYYY**GSGRRGDLS as the most amyloidogenic regions, respectively (Figure 2A). The ArchCandy predictor also returned a predicted fibril architecture that involves the three contiguous tyrosines of the EYYY motif in the first β-strand of β-strand-loop-β-strand motifs (Figure 4C). The prediction of ArchCandy relies on the assumption that protein sequences which are able to form β-arcades are amyloidogenic. Indeed, the core structural element of a majority of naturally-occurring and disease-related amyloid fibrils is a β-arcade, representing a parallel and in-register stacks of β-strand-loop-β-strand motifs called β-arches [100].

Taking into account the role of aromatic residues in promoting the homotypic aggregation of aggregation-prone regions (APRs) via β-strand interactions [101], we targeted the triple tyrosine motif for mutagenesis and replaced these tyrosines with three alanines to yield the PNT3^3A^ variant. All three used computational predictors, suggesting that these mutations will decrease the amyloidogenicity of the native PNT3 protein. The purified PNT3^3A^ variant (Figure 2A) lost the ability to form macroscopically visible, phase-separated condensates that bind CR (Figure 4A). CR shift assays confirmed that the variant has a reduced ability to bind CR, although it still binds the dye more than the NTail control (Figure 4B). Finally, TEM studies showed that the variant has a significantly reduced ability to form amyloid-like fibrils (Figure 4D). As expected, in light of the much-reduced ability of PNT3^3A^ to form fibrils, only a moderate signal decrease was observed in the CD spectrum of the variant following a 24-h incubation at 37 °C compared to PNT3 (cf. Figure 5A,B).

### 3.8. CR-Staining of Transfected and Infected Mammalian Cells

**Figure 11:**
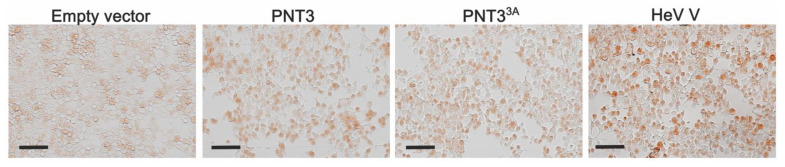
CR-staining of HEK 293T cells transfected with an empty vector or with constructs driving the expression of PNT3 or PNT3^3A^, or V at 48 h post transfection. Scale bar: 200 μm.

Taking into account the fact that virtually every protein can phase-separate and form amyloid-like fibrils, provided that a sufficiently wide range of experimental conditions is explored [85], we reasoned that in vitro fibril formation by PNT3 might merely reflect this general property of proteins and hence be devoid of functional relevance. As a first step towards the assessment of the ability of PNT3 to also form fibrils in a cellular context, we carried out transfection experiments of HEK 293T cells. CR-staining experiments showed that cells transfected to express PNT3 capture CR more than cells transfected with an empty vector (Figure 11), providing clues about the formation of PNT3 fibrils also in a cellular context. Surprisingly, cells transfected with a construct driving the expression of the PNT3^3A^ variant that has a significantly lower ability to form fibrils in vitro still appeared to be able to capture CR, although the intensity of the staining appeared to be slightly lower compared to PNT3 transfected cells (Figure 11). The difference in staining between PNT3 and PNT3^3A^ transfected cells could be better appreciated when cells where stained 72 h after transfection (Supplementary Figure S5). In this case, indeed, cells transfected to express the variant appeared clearly less stained (Supplementary Figure S5), although at this late stage of transfection, much fewer cells were present, possibly due to the cytotoxicity of the overexpressed protein that resulted in the cell detachment. Note that the immunofluorescence analysis ruled out the possibility that the reduced CR capture by PNT3^3A^ transfected cells might have arose from a reduced expression of the mutated protein (Supplementary Figure S6A–C).

Worthy to note, cells transfected to express the full-length V protein were also found to capture CR (Figure 11 and Supplementary Figure S5). Notably, they even appeared to capture more CR than cells transfected by the PNT3 construct. The immunofluorescence analysis of cells transfected by the V construct (see Supplementary Figure S6D) revealed that the V protein was expressed at levels comparable to those of PNT3 (and PNT3^3A^). The more pronounced CR capture by cells expressing V might thus reflect a higher intrinsic fibrillation propensity of the full-length protein compared to its PNT3 region, a possibility that will be explored in future studies.

**Figure 12:**
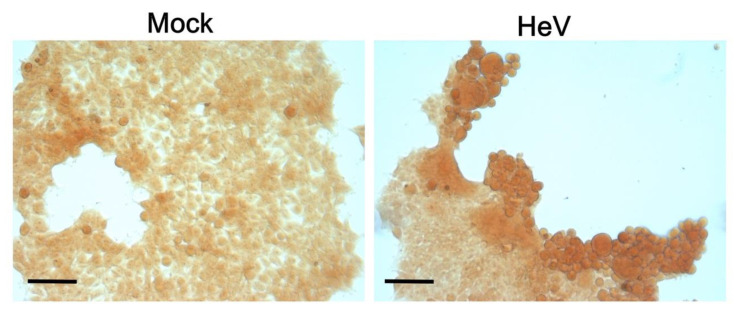
CR capture by non-infected HEK 293T cells (Mock) or by cells infected by HeV (MOI 0.00025) at 48 post-infection. Scale bar: 100 μm.

Of even more relevance, CR capture was also observed in the case of cells infected with HeV (Figure 12). As shown in Figure 12, infected cells were much more stained at 48 h p.i. than non-infected (mock) cells. Note that we chose to use a very low MOI (0.00025) to limit the very high cytopathic effects of HeV. As a result, not all cells were infected and hence not all cells captured CR. In further support of a biologically relevant phenomenon, syncytia induced by the co-expression of HeV F and G proteins in HEK 293T cells captured a very low amount of CR compared to the infected cells, thus ruling out the possibility that CR capture can merely reflect a cellular stress consecutive to the formation of virus-induced syncytia (Supplementary Figure S6E).

We further confirmed the observations made in the infected HEK293T cells and also in human pulmonary microvascular endothelial cells (HPMEC), which are prototypic of one of the main primary targets of Henipavirus infection (Supplementary Figure S7). Cells were infected at the same MOI and both fixation and staining were performed 48 h after infection. Here again, syncytia, which reflects the most infected cells, were found to capture significantly more CR than other cells (Supplementary Figure S7).

Altogether, these data provide hints supporting the formation of amyloids-like fibrils also in cellula.

## 4. Discussion

In this paper we showed that the HeV V protein undergoes a liquid-to-hydrogel transition and mapped the region responsible for this behavior to the long IDR of V and specifically to residues 200–310 (PNT3). PNT3 was shown to phase-separate in vitro into solid-like condensates, as shown by FRAP. These condensates bind the amyloid-specific dye CR and TEM revealed that PNT3 forms amyloid-like fibrils. Interestingly, while PNT3 was found to bind CR, thioflavin T-binding assays yielded erratic and non-reproducible results. In this regard, it is noteworthy that some amyloid-like fibrils, such as those formed by the fused-in-sarcoma RNA-binding protein (FUS) [102] or by the Japanese variant of Aβ [103], bind poorly (if at all) to thioflavin T, although, so far, no study has investigated the molecular basis of this behavior in detail. Additionally, vice versa, amyloids that are not stainable with CR have been described as well [104].

The behavior of the rationally designed PNT3^3A^ variant, which was found to have a much reduced ability to form fibrils, provides insights into the nature of the interactions driving fibril formation and their architecture, and enables identifying the YYY motif as being likely part of a hydrophobic core. Converesely, the fact that the substitution of the triple tyrosine motif only reduces but does not fully abrogate the ability of PNT3 to form amyloid-like fibrils advocates for a scenario whereby other motifs and/or sequence attributes, that remain to be identifed, contribute to fibrillation. At the same time, it provides hints pointing to the ability of the triple alanine motif to still contribute to the formation of a hydrophobic core, although the latter appears to be much less stable compared to that formed by the *wt* protein.

Gelation can occur with or without phase separation [105]. In gelation driven by phase separation, multivalent proteins condense into dense droplets and gels form within droplets. In this case, proteinaceous condensates resulting from LLPS are referred to as undergoing “maturation” towards a gel or solid state, the phenomenon being referred to as phase transition [38,41,43,75]. However, gels can also form without a condensation or demixing of proteins into droplets. Gelation driven by phase separation requires lower protein concentrations and seems to be the biologically preferred mechanism for forming membraneless organelles [105].

Phase-separated condensates can nucleate amyloid-like fibrils, the nucleation process being enhanced in droplets or gels due to local concentration increase effects. A recent example is provided by the SARS-CoV2 nucleocapsid protein that forms amyloids in phase-separated droplets [106]. In line with this, IDRs are known to form amyloid-like structures via the formation of hydrogels [87,107,108]. Additionally, vice versa, amyloid-like fibers are also known to be able to form highly stable hydrogels [67,109]. As such, gelation and fibrillation appear to be intertwined, but understanding which process is the trigger and which is the result remains difficult. To add an additional layer of complexity, depending on the conditions, amino acid sequence, and post-translational modifications, a given protein can condense into different high-order structures, such as liquids, glassy solids, or amyloid fibers, whose molecular dynamics, internal organization, interaction strength, and reversibility are distinct [38,75,110]. Although liquid-to-solid transitions can be functional (see [111] for an example), an increasing number of evidences point to liquid-to-hydrogel transitions as the underlying pathological protein aggregation associated with neurodegenerative diseases [44,112].

In the present study, we have primarily documented fibril formation by PNT3 without exploring the early stage of the process or the precise relationships between fibrillation and gelation. Future efforts will investigate the early steps and the kinetics of the process. In addition, future efforts will attempt to disentangle the complexity of the system both by ascertaining whether solid-like condensates result from the maturation of short-lived droplets resulting from LLPS and by establishing to which extent phase separation accelerates fibril formation.

Irrespective of the mechanism driving fibrils formation, it is tempting to speculate that the ability of PNT3 to form amyloids may constitute at least one of the possible molecular mechanisms underlying the pathogenicity of HeV. Worthy to note, the CR-staining experiments herein provide hints suggesting that PNT3 fibrils not only form in vitro but also in the cellular context. In fact, cells transfected to express PNT3 captured CR. Most importantly, cells infected with HeV were positive to CR-staining as well, indicating that the formation of amyloid-like structures does not arise from the typically higher protein expression levels achieved upon transfection but also occurs in the context of infection. Although we cannot formally rule out the possibility that CR-staining may arise from virus-induced fibrillation of cell components, either alone or in combination with viral proteins, the fact that transfected cells expressing PNT3 captured CR does not advocate in favor of this scenario. CR-capture by infected cells can arise from fibrils that are formed by the V, W, or P protein (or a combination of them): indeed, considering all these proteins share the NTD and hence the PNT3 region, it is conceivable that they all can undergo fibrillation. In line with these expectations, we have recently shown that the W proteins form amyloid-like fibrils in vitro and in fibrillar aggregates in the nuclei of transfected cells [21]. Future studies will assess the ability of the P protein to fibrillate and establish possible synergistic contributions to the PNT3 fibrillation that are brought by the CTD of these proteins and, in particular, by the P multimerization domain that might enhance this process through multivalency, a key property in phase separation (see [113] and references therein cited).

What could be the functional impact of fibril formation? The formation of amyloids by viral proteins is a rather poorly explored field, with very few examples having been reported so far. The first reported example pertains the human papilloma virus (HPV)-16 E7 protein (i.e., the major oncoprotein of HPV). HPV E7 was indeed shown to be able to self-assemble into defined spherical oligomers with amyloid-like properties [114,115], although the possible functional implications of this phenomenon were only discussed in terms of the amyloid-cancer connection [116] and not in relation with the viral disease. After this first study, PB1-F2 from the influenza A virus was reported to form amyloids [117]. PB1-F2 is an intrinsically disordered accessory protein involved in virulence by inducing mitochondria-mediated immune cells’ apoptosis. Amyloids of PB1-F2 were shown to disrupt cell membranes both when added to cells and in infected cells, and to be highly cytotoxic [117]. A link was established between the formation of amyloid-like assemblies and the membrane-lytic activity of the protein, thereby contributing to shed light on the mechanisms underlying amyloid toxicity. In subsequent studies (reviewed in [118]), the ability of viral proteins to form fibrillar aggregates was shown to be associated with various functional effects such as the blockade of necroptosis or apoptosis via the formation of hybrid amyloids with host-cell amyloids (RHIM-containing proteins in members of the *Herpesviridae* family) [119,120,121] and the blockade of the stress granule assembly (VP35 from Ebola virus) [122]). In addition, fibrillar aggregates made of the NSs protein from Rift Valley Fever virus were found to suppress host cell RNA synthesis through host transcription factors’ sequestration [123]; later on, a functional link was established between the formation of NSs amyloids and virulence, in which NSs fibrils were shown to suppress IFN responses through the silencing of IFN-β expression and the degradation of PKR [124].

In light of the functional role of the V and W proteins in counteracting the IFN-mediated host innate immune response, it is tempting to hypothesize that the fibrillar aggregates, driven by the PNT3 region and formed in infected cells, might sequester key cell proteins involved in the antiviral response. Future efforts will be devoted to identifying host proteins interacting with PNT3-driven fibrils and to unravelling the functional impact of fibril formation in the cellular context.

The relative fragility of PNT3 fibers, pinpointed by their sensitivity to SDS, might reflect a role as regulatory switches, i.e., fibers can form and disassemble in response to changes in the surrounding environment and this can play a role in the (in)activation of specific cellular pathways [38,40,125]. If fibers result from the maturation of droplets resulting from LLPS, then their ability to disassemble might impart metastability (i.e., reversibility) to membrane-less organelles containing these amyloidogenic proteins. Given the link between material properties and pathological conditions [85,126], cells have evolved mechanisms to monitor and control the fluidity of phase-separated droplets. Consistent with this, many ribonucleoprotein (RNP) bodies and granules are enriched in ATP-dependent chaperones such as Hsp70 and Hsp40 [127]. In agreement, the viscoelasticity of nucleoli and the dynamics of stress granule components exhibit a strong ATP dependence [128,129]. Our results showing that Hsp70 impairs fibril formation by PNT3 are in line with these previous findings and may reflect a mechanism whereby variations in Hsp70 intracellular levels, typically occurring during viral infections (see [130] and references therein cited), may control the efficiency of the formation of fibrils containing the P/V/W protein. In addition, the ability of Hsp70 to hamper fibril formation by PNT3 may also be linked to the well-documented protective role of this chaperone against (paramyxo)viral infections, for which Hsp70-mediated enhancement of virus transcription and replication ultimately was paradoxically found to contribute to virus clearance through the stimulation of both innate and adapatative immune responses [130,131,132,133,134].

In *Mononegavirales*, transcription and replication take place in viral factories, e.g., cytoplasmic inclusions in which viral replication and assembly take place, and where specific viral and cellular proteins, along with nucleic acids, concentrate. They serve as platforms for optimized viral replication via selective uptake or the exclusion of components and shielding from the host immune defense [135,136,137,138,139,140,141]. In many *Mononegavirales* members, viral factories were shown to have liquid properties and to result from LLPS of their N and P proteins [118,142,143,144,145,146,147,148,149], with IDRs playing a critical role in the process [118]. As already mentioned, the minimal region of HeV V, conferring the ability to phase separate (i.e., PNT3), is also part of the P protein that is a constituent of the viral factories of henipaviruses [150]. It is conceivable that the ability of PNT3 to phase separate may also be functionally coupled to the formation of viral factories. As the P protein is an essential component of the replicative complex of *Mononegavirales* and considering the presence of large IDRs is a widespread and conserved property in *Mononegavirales* P proteins [151,152,153,154,155,156,157,158,159,160,161,162,163,164,165], the present results promise to have broad implications for a large number of important human pathogens.

## 5. Conclusions

The present study provides an additional example, among the few reported so far, of a viral protein forming amyloid-like fibrils, thereby significantly contributing to enlargement of our currently limited knowledge of viral amyloids. It also constitutes an asset for future research avenues that will tackle the functional impact of fibrils formation in terms of virus-induced cytopathic effects and host innate immune response evasion. In this regard, the availability of the PNT3^3A^ variant, which has a much-reduced fibrillation ability, is a valuable tool as it opens up the possibility of establishing possible functional links between fibrillation and virulence.

## Figures and Tables

**Table 1:** SAXS and SEC-SAXS data acquisition parameters.

Experiment Type and Aim	SAXS Aggregation Process	SEC-SAXS Conformational Studies
**Data acquisition**		
Instrument	European Synchrotron Radiation Facility (Grenoble, France) Beamline BM29	SOLEIL Synchrotron (Gif-sur-Yvette, France) Beamline Swing
X-rays wavelength (Å) Energy (keV)	0.992 12.5	1.033 12
Detector type	Pilatus 1M	Dectris EIGER 4M
Sample-to-detector distance (m)	2.847	2.0
q-range	0.028–4.525 nm^−1^	0.0036–0.5397 Å^−1^
Temperature (°C)	20	
**Samples**		
Concentration (mg mL^−1^)	1.0 and 2.0	3.5
Sample volume (µL)	50	70
Gel filtration column Flow rate (mL min^−1^)	-	BioSec 3-300 (Agilent) 0.2
Buffer	50 mM sodium phosphate pH 6.5, 5 mM EDTA (buffer C)	

## Data Availability

The data present in the current study are available from the corresponding author upon reasonable request.

## Supplementary Materials

The following are available online at https://www.mdpi.com/article/10.3390/biom11091324/s1, Figure S1: Mass spectrometry analysis of PNT3 and PNT3^3A^; Figure S2: SAXS studies of PNT3 at 1 mg mL^−1^; Figure S3: SEC-SAXS studies of PNT3; Figure S4: Sensitivity to SDS of PNT3 fibers as a function of time; Figure S5: CR-staining of transfeced cells; Figure S6: Fluorescence analysis of transfected cells and CR-staining of late syncytia induced by the transfection with constructs encoding F and G; Figure S7: CR-staining of HPMEC cells, either non-infected or infected by HeV; Supplementary Table S1: List of primers used to generate the various constructs; Supplementary Table S2: LLPS propensities as provided by various predictors for the full-length HeV and NiV V proteins, as well as for the HeV NTD and its PNT3 region; Supplementary Video S1: Movie of the brightfield of the PNT3 condensate analyzed by FRAP in Figure 3C; and Supplementary Video S2: Movie of the AF488-labeled PNT3 condensate analyzed by FRAP in Figure 3C.
