# Supplementary material for: Identification of a Region in the Common Amino-terminal Domain of Hendra Virus P, V, and W Proteins Responsible for Phase Transition and Amyloid Formation
Source: Biomolecules. 2021 Sep 7;11(9):1324. doi: 10.3390/biom11091324 (PMC8471210; doi:10.3390/biom11091324)
Supplement: Supplementary file 1 [file biomolecules-11-01324-s001.zip › biomolecules-1336844-supplementary-for final.pdf]

Identification of a Region in the Common Amino-terminal Domain of Hendra virus P, V, and W Proteins Responsible for Phase Transition and Amyloid Formation

Edoardo Salladini [1], Frank Gondelaud [1,†], Juliet F. Nilsson [1,†], Giulia Pesce [1,†], Christophe Bignon [1], Maria Grazia Murrali [2], Roxane Fabre [3], Roberta Pierattelli [2], Andrey V. Kajava [4], Branka Horvat [5], Denis Gerlier [5], Cyrille Mathieu [5] and Sonia Longhi [1,*]

1 Laboratory Architecture et Fonction des Macromolécules Biologiques (AFMB), UMR 7257, Aix Marseille University and Centre National de la Recherche Scientifique (CNRS), 13288 Marseille CEDEX 9, France; edoardo.salladini@gmail.com (E.S.); frank.gondelaud@univ-amu.fr (F.G.); juliet.nilsson@univ-amu.fr (J.F.N.); giulia.pesce@univ-amu.fr (G.P.); christophe.bignon@univ-amu.fr (C.B.)
2 Magnetic Resonance Center (CERM) and Department of Chemistry "Ugo Schiff", University of Florence, 50019 Sesto Fiorentino, Italy; mgmurrali@gmail.com (M.G.M.); roberta.pierattelli@unifi.it (R.P.)
3 Centre d'Immunologie de Marseille-Luminy (CIML), Aix Marseille University, CNRS, Institut National de la Santé et de la Recherche Médicale (INSERM), 13288 Marseille CEDEX 9, France; fabre@ciml.univ-mrs.fr
4 Centre de Recherche en Biologie Cellulaire de Montpellier, UMR 5237, CNRS, Université Montpellier; Montpellier, France; andrey.kajava@crbm.cnrs.fr
5 Centre International de Recherche en Infectiologie (CIRI), Team Immunobiology of the Viral infections, Univ Lyon, INSERM, U1111, CNRS, UMR 5308, Université Claude Bernard Lyon 1, Ecole Normale Supérieure de Lyon, 69007 Lyon, France; branka.horvat@inserm.fr (B.H.); denis.gerlier@inserm.fr (D.G.); cyrille.mathieu@inserm.fr (C.M.)
* Correspondence: Sonia.longhi@univ-amu.fr
† These authors contributed equally to this work.

Supplementary Figures S1 to S7
Supplementary Table S1 and S2

Supplementary Video S1: Movie of the brightfield of the PNT3 condensate analyzed by FRAP in Figure 3C,D.

Supplementary Video S2: Movie of the AF488-labeled PNT3 condensate analyzed by FRAP in Figure 3C,D.

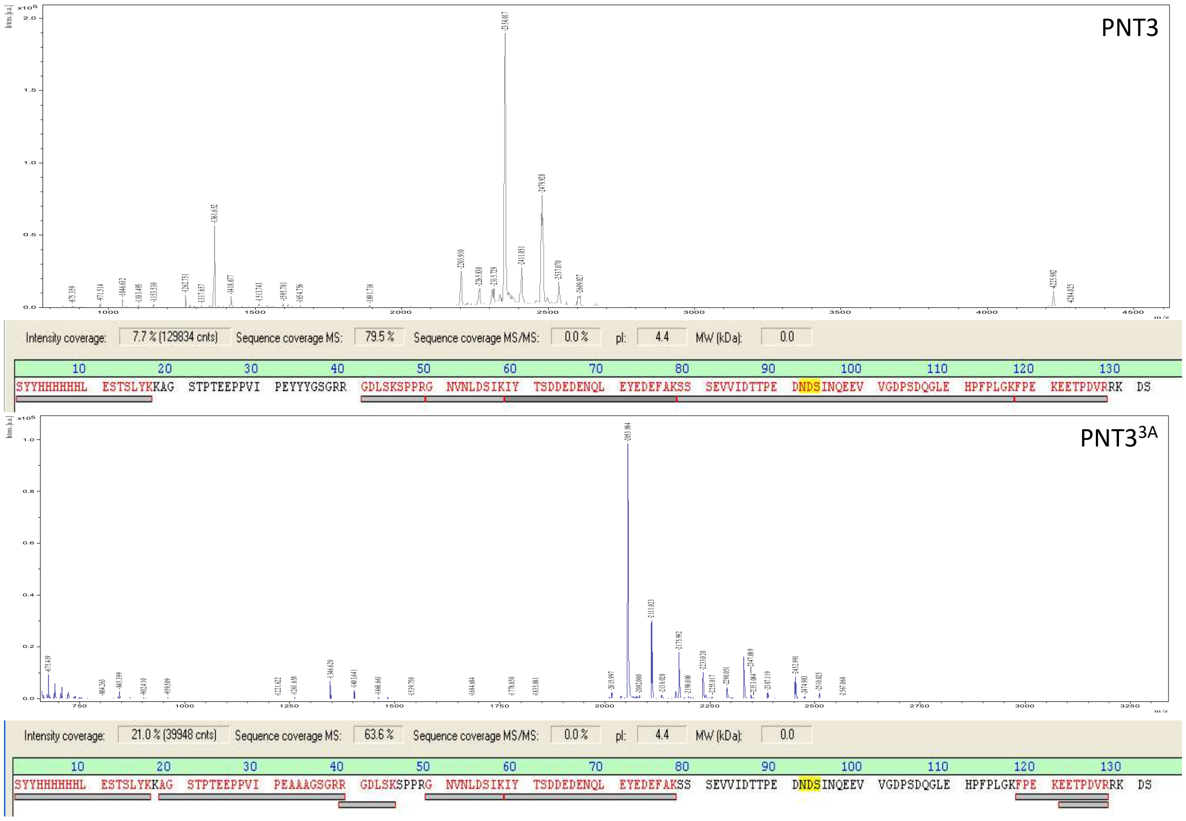

Figures S1. Mass spectrometry analysis of PNT3 (**A**) and PNT3^{3A} (**B**). The band of the protein was excised from an SDS acrylamide gel and then subjected to trypsin digestion. A coverage of 79.5% and 64% of the sequence was obtained for PNT3 and PNT3^{3A}, respectively. Results are consistent with the initial methionine having been cleaved off. Peptides identified in the MS spectrum are highlighted by gray bars, with a color gradient ranging from light to dark gray with increasing peak intensities.

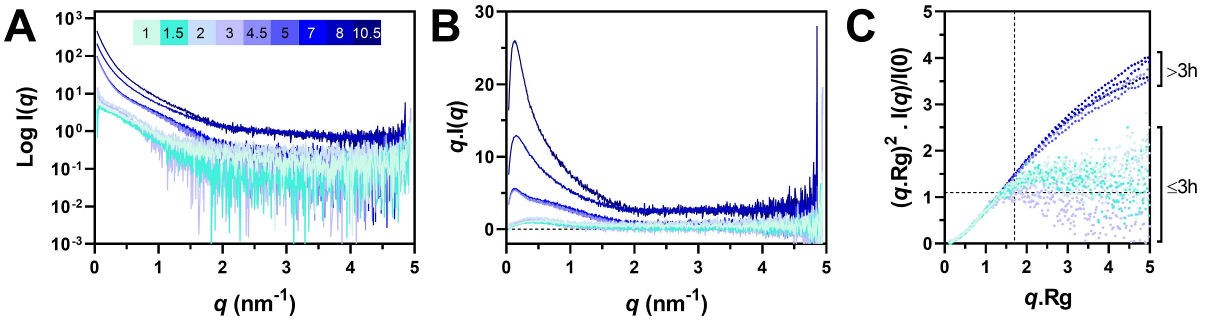

Figures S2. The PNT3 aggregation process followed by SAXS at a concentration of 1 mg mL^{-1}. (**A**) Scattering intensities of PNT3 recorded at various time-points (from 1 h to 10.5 h) of incubation at 37°C. (**B**) Total scattered intensities. The color code is the same as in (A). (**C**) Normalized Krakty plots. The black dotted lines indicate the maximum of a bell-shaped curve as observed for globular proteins. The color code is the same as in (A) and (B).

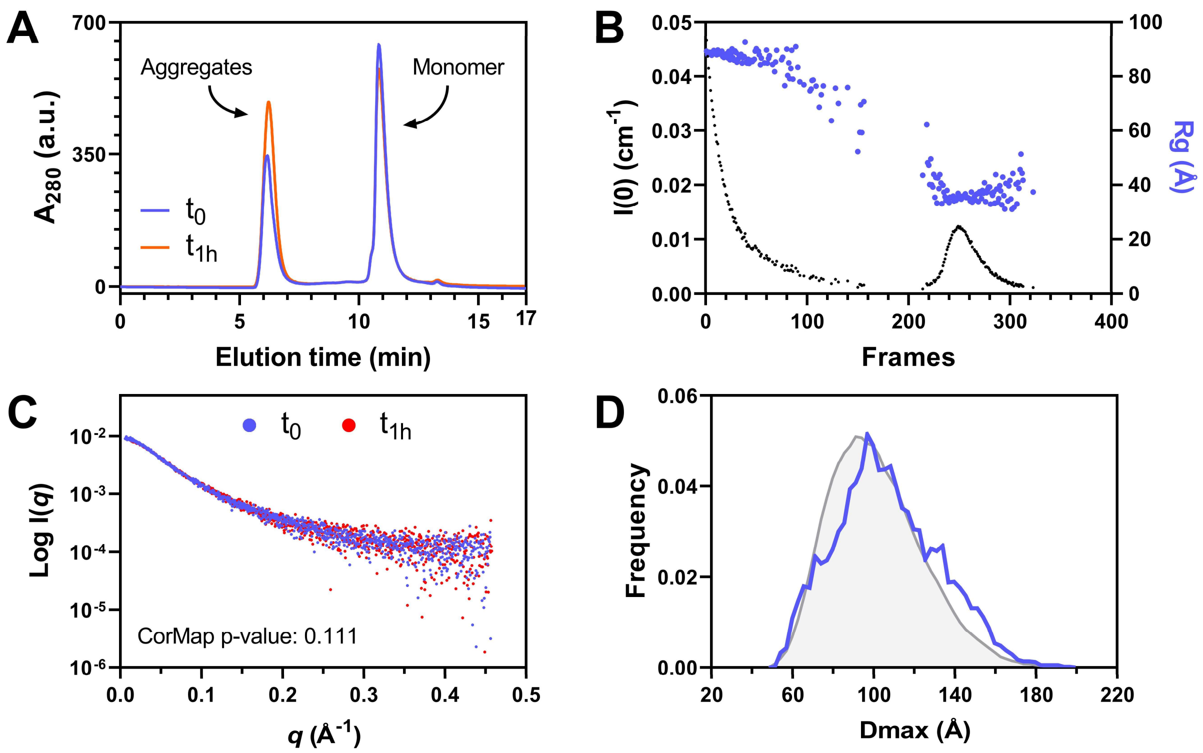

Figures S3. SEC-SAXS analysis of the monomeric form of PNT3. (**A**) Far-UV chromatogram as obtained after the injection of PNT3 on a BioSec 3-300 (Agilent) column during the SEC-SAXS experiments. (**B**) *I*(0) and R_g estimation as a function of the recorded frames as obtained after the injection of PNT3 at time zero on a BioSec 3-300 (Agilent) column during the SEC-SAXS experiments. (**C**) Comparison of the final scattering intensities after the data treatment of PNT3 after 1 h (red) and zero h (blue) of incubation at 37 °C. The CorMap p-value is indicated. (**D**) D_{max} distribution of the selected ensemble of PNT3 (blue) and of the initial pool of random coil conformers (gray).

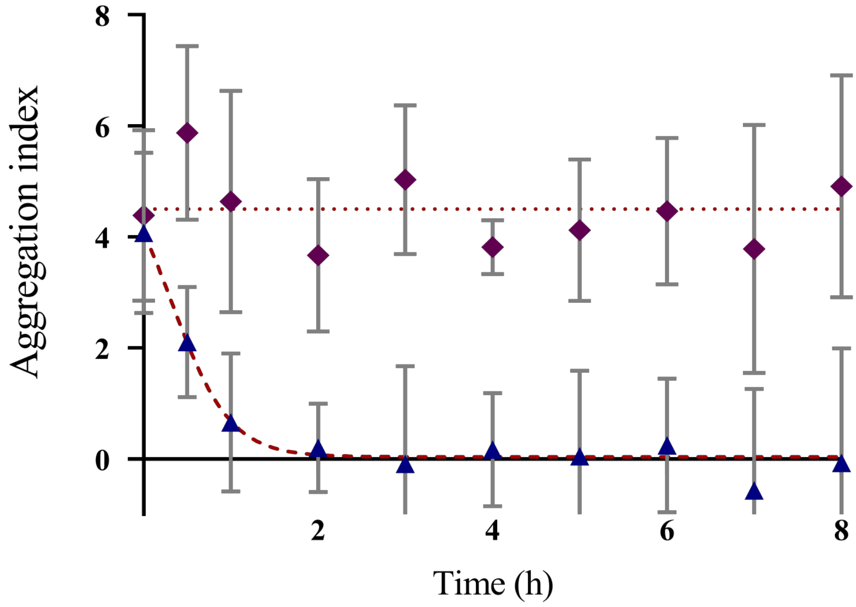

Figures S4. Aggregation index of a fibrillated PNT3 sample (as obtained after incubation for 72 h at 37 °C) as a function of the time (hours) of incubation at RT either in the absence or in the presence of 2% SDS. The protein concentration was 100 μM. The error bar corresponds to the standard deviation with n = 5.

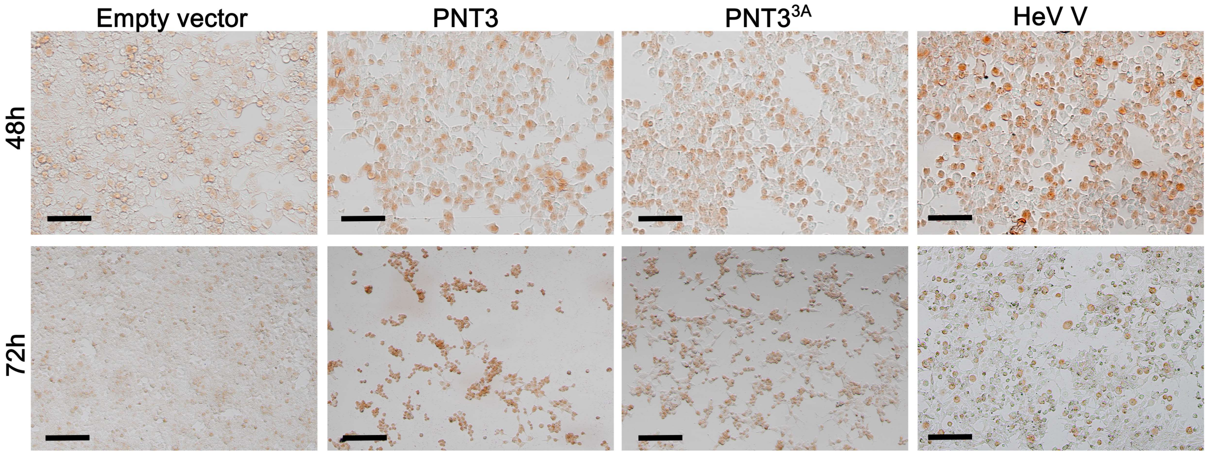

Figures S5. CR-staining of HEK 293T cells transfected with an empty vector or with constructs driving the expression of PNT3 or PNT3^{3A}, or V at 48 h and 72 h post-transfection. Note the drastic reduction in the number of cells at 72 h likely due to the cytotoxicity of the overexpressed protein, resulting in cell detachment. Scale bar: 200 µm.

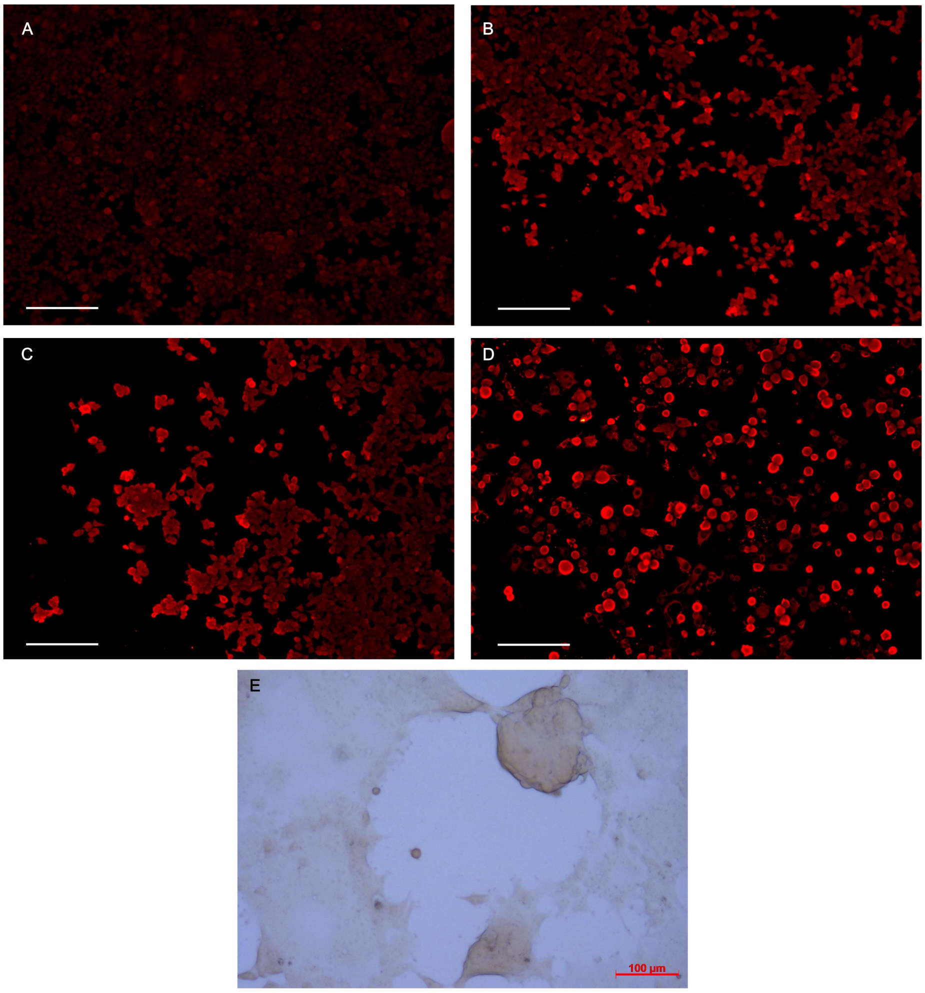

Figures S6. (A-D) Immunofluorescent staining of HEK 293T cells transfected with an empty vector (**A**) or with constructs driving the expression of PNT3 (**B**), PNT3^{3A} (**C**), or V (**D**) using a Penta-His HRP conjugate. Scale bar: 500 µm. (**E**) CR-staining of late syncytia induced 48 h after co-transfection of HEK cells with constructs driving the expression of the HeV F and G proteins.

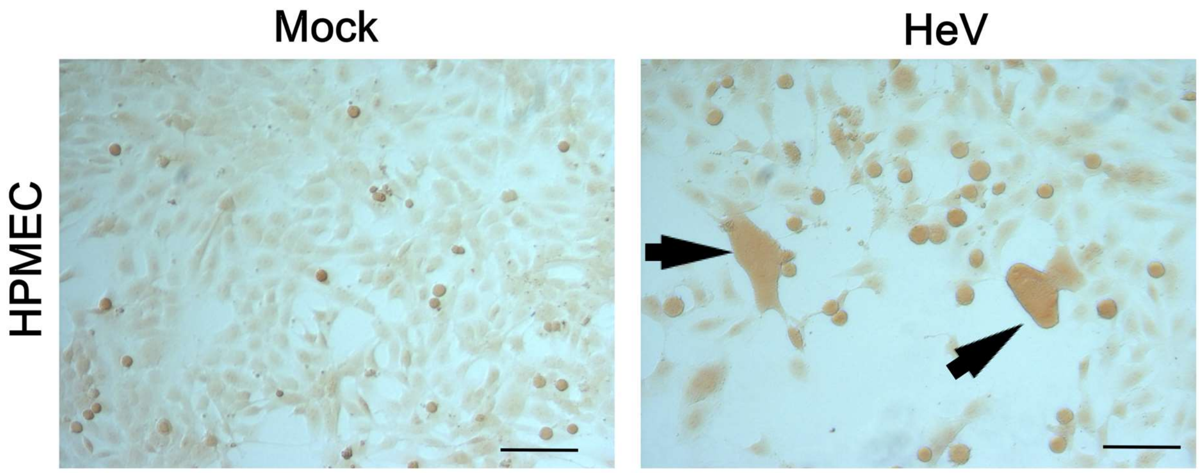

Figures S7. CR capture by non-infected (mock) or HPMEC cells infected by HeV (MOI 0.00025) at 48 h post-infection. The black arrows show large syncytia formed between the infected cells. Scale bar: 100 µm.

Table S1. Nucleotide sequence of the primers used to generate the various constructs.

Primer name	Sequence (5'-3')	Name of the construct
PNT1-AttB1	GGGGACAAGTTTGTACAAAAAAGCAGGCTTCGATAAACTG-GATCTGGTTAACG	PNT1-pDEST17
PNT1-AttB2	GGGGACCACTTTGTACAAGAAAGCTGGGTCTTATTACATCG-GATCCAGCTGGATATC	
PNT2-AttB1	GGGGACAAGTTTGTACAAAAAAGCAGGCTTCGAAGATCCGGATGA-TATCCAG	PNT2-pDEST17
PNT2-AttB2	GGGGACCACTTTGTACAAGAAAGCTGGGTCTTATTATTCCGGAATAAC-CGGCGGTTC	
PNT3-AttB1	GGGGACAAGTTTGTACAAAAAAGCAGGCTTCACCCCGACCGAAGAAC-CGCCG	PNT3-pDEST17
PNT3-AttB2	GGGGACCACTTTGTACAAGAAAGCTGGGTCTTATTAGCTATCTTTACGAC-GCACATC	
PNT4-AttB1	GGGGACAAGTTTGTACAAAAAAGCAGGCTTCGAAGAAACCCCG-GATGTGCGT	PNT4-pDEST17
PNT4-AttB2	GGGGACCACTTTGTACAAGAAAGCTGGGTCTTATTATTTTTGATCGG-CATAATACGGC	
B1HisNT3	GTACAAAAAAGCAGGCTCGCATCACCACCATCACCATACCCCGACCGAA-GAACCGCC	His6-PNT3-pTH31
NT3B2	ACCACTTTGTACAAGAAAGCTGGGTcGCTATCTTTACGACGCACATC AC-CTGTTCGTTGCAACAAATTGATGAG-	
attL1a	CAATGCTTTTTTATAATGCCAAGTTTGTACAAAAAAGCAGGC TTTT-GACTGATAGTGACCTGTTCGTTGCAACAAATTGATAA-	
attL2a	GCAATGCTTTCTTATAATGCCCACTTTGTACAAGAAAGCTGGG	
HindNT3	GCGTTTAAACTTAAGCTTCCACCATGGCTAGCGATCAAACAAG	*His-PNT3-GFPq-pCDNA3.1+
NT3R	GGTGGCGACCGGTACCCATCG	
GFPqF	CGATGGGTACCGGTCGCCACCATGGCTAGCAAAGGAGAAG	
GFPqXhoI	GGGCCCTCTAGACTCGAGTTATCAGTTGTACAGTTCATCC	
HindNT3	GCGTTTAAACTTAAGCTTCCACCATGGCTAGCGATCAAACAAG	His-PNT3-pCDNA3.1+
NT3XhoI	TTAAACGGGCCCTCTAGACTCGAGTTATTAGGTGGCGACCGG-TACCCATCG	
attB1	ACAAGTTTGTACAAAAAAGCAGGCT	PNT3^{3A}-pDEST17
R_3ala-pNT3	TCGCCACGACGGCCGCTACCGGCTGCCGCTTCCGGAATAAC-CGGCGGTTCTTC	
F_3ala-pNT3	AACCGCCGGTTATTCCGGAAGCGGCAGCCGG-TAGCGGCCGTCGTGGCGATCTG	
attB2	ACCACTTTGTACAAGAAAGCTGGGT	
HindNT3	GCGTTTAAACTTAAGCTTCCACCATGGCTAGCGATCAAACAAG	His-PNT3^{3A}-pCDNA3.1+
R_3ala-pNT3	TCGCCACGACGGCCGCTACCGGCTGCCGCTTCCGGAATAAC-CGGCGGTTCTTC	

F_3ala-pNT3	AACCGCCGGTTATTCCGGAAGCGGCAGCCGG-TAGCGGCCGTCGTGGCGATCTG	
NT3XhoI	TTAAACGGGCCCTCTAGACTCGAGTTATTAGGTGGCGACCGG-TACCCATCG	

* For the eukaryotic expression of PNT3-GFP (unpublished and used in the present study as a PCR template only), another His6-tagged PNT3-GFP construct was generated by PCR using a GFP coding sequence optimized for eukaryotic expression. In the first PCR, the coding sequence of PNT3 was amplified using His6-PNT3-pTH31 as a template and as primers, namely HindNT3 (GCGTTTAAACTTAAGCTTCCACCATGGCTAGCGATCAAACAAG) and NT3R (GGTGGCGAC-CGGTACCCATCG). In the second PCR, the coding sequence of GFP was PCR-amplified using GFPq in pTTo (Durocher et al., Nucleic Acids Res 2002, PMID: 11788735) as a template and as primers, namely GFPqF (CGATGGGTAC-CGGTCGCCACCATGGCTAGCAAAGGAGAAG) and GFPqXhoI (GGGCCCTCTAGACTCGAGTTATCAGTT-GTACAGTTCATCC). After DpnI treatment, 1 µL of the PCR1 and 1 µL of the PCR2 were used as overlapping megaprimers, along with primers HindNT3 and GFPqXhoI in a third PCR. After purification, the third PCR product was digested by HindIII and XhoI, and ligated to pCDNA3.1+ that had been digested with the same enzymes. The construct was called His-PNT3-GFPq-pCDNA3.1+.

Table S2. LLPS propensities as provided by various predictors for the full-length HeV and NiV V proteins, and for the HeV N-terminal domain and its PNT3 region. Scores reflecting a significant propensity to undergo LLPS are shown in red.

	**PSpredictor**	**catGranule**	**FuzPred**
**HeV V (aa 1–457)**	0.86	1.16	0.99
**HeV NTD (aa 1–404)**	0.96	1.38	0.99
**HeV PNT3 (aa 200–310)**	0.17	0.46	0.88
**NiV V (aa 1–456)**	0.52	1.01	0.90
